# Role of N6-methyladenosine methylation in transverse aortic constriction-induced cardiac fibrosis: insights from MeRIP-seq analysis

**DOI:** 10.1007/s11033-025-10940-2

**Published:** 2025-08-26

**Authors:** Shidong Liu, Hongxu Liu, Qiyuan Bai, Zhili Wei, Pengying Zhao, Hao Chen, Bing Song, Cuntao Yu

**Affiliations:** 1 The First Clinical Medical College of Lanzhou University, Lanzhou, China; 2 Department of Cardiovascular Surgery, First Hospital of Lanzhou University, Lanzhou, China; 3 Department of Cardiovascular Surgery, Fuwai Hospital, National Center for Cardiovascular Diseases, Chinese Academy of Medical Sciences, Peking Union Medical College, Beijing, China

**Keywords:** Methylated RNA immunoprecipitation sequencing, Transverse aortic constriction, Cardiac fibrosis, N6-methyladenosine

## Abstract

This study aimed to investigate the role and potential mechanisms of N6-methyladenosine (m6A) methylation in a mouse model of transverse aortic constriction (TAC)-induced cardiac fibrosis using MeRIP-seq. A TAC-induced cardiac fibrosis mouse model was established, and cardiac function and structural parameters were assessed by echocardiography four weeks post-surgery. The global m6A methylation levels in myocardial tissues were evaluated using Dot blot analysis, and the expression levels of m6A-modifying enzymes (METTL3, METTL14, ALKBH5, FTO) were detected by qPCR and Western blot. Additionally, methylated RNA immunoprecipitation sequencing (MeRIP-Seq) was performed to identify differentially methylated sites, followed by Gene Ontology (GO) and Kyoto Encyclopedia of Genes and Genomes (KEGG) pathway enrichment analyses to determine the biological functions and signaling pathways of m6A-methylated genes. The results showed that TAC surgery successfully induced a cardiac fibrosis model in mice, as indicated by significantly reduced EF and LVEF and increased LVIDd and LVIDs. Moreover, m6A methylation levels were elevated in myocardial tissues of TAC mice, accompanied by upregulation of METTL3 and METTL14 expressions and downregulation of ALKBH5 and FTO expression. MeRIP-seq revealed that m6A peaks were primarily enriched in the 3’ UTR regions, with 1,466 differentially methylated sites identified between TAC and sham groups, including 717 hypermethylated and 749 hypomethylated sites. Functional enrichment analyses showed that these differentially methylated genes were involved in various biological processes, including signal transduction, transcriptional regulation, and ion channel activity, and were associated with pathways such as type 2 diabetes mellitus, signaling pathways regulating pluripotency of stem cells, and insulin signaling. Thus, our findings suggested that m6A methylation played a significant role in TAC-induced cardiac fibrosis by regulating key genes involved in myocardial remodeling and functional impairment.

**Supplementary Information:**

The online version contains supplementary material available at 10.1007/s11033-025-10940-2.

## Introduction

Cardiac fibrosis, characterized by excessive deposition of extracellular matrix (ECM) components, is a key pathological feature of various cardiovascular diseases, including hypertensive heart disease and myocardial infarction [1, 2]. The progressive fibrotic remodeling of the myocardium impairs cardiac function, leading to significant morbidity and mortality [3, 4]. In recent years, epigenetic modifications have attracted considerable attention within the field of cardiovascular diseases [5]. Among these modifications, N6-methyladenosine (m6A) methylation has emerged as a significant RNA modification that plays a crucial role in regulating gene expression. m6A methylation influences various biological functions by modulating RNA stability, splicing, translation, and degradation [6]. Previous studies have demonstrated that alterations in m6A RNA methylation contribute to the progression of heart failure by affecting translation [7–9]. A separate study examined the dynamic patterns of m6A profiles in mRNA associated with cardiomyocyte regenerability during early cardiac development in mice. This investigation demonstrated that m6A methylation is connected to the heart’s proliferative and regenerative capacities shortly after birth [10]. Furthermore, the involvement of m6A methylation in myocardial ischemia-reperfusion injury and doxorubicin-induced cardiotoxicity has been explored. The findings indicate that m6A methylation influences the pathophysiological processes of these conditions by modulating cellular autophagy, apoptosis, oxidative stress, and inflammatory responses [11]. One study has shown that METTL3, an m6A methyltransferase, was significantly upregulated in fibrotic cardiac tissues and cardiac fibroblasts [12]. This upregulation was associated with increased m6A methylation of specific mRNAs, which promoted the activation and proliferation of cardiac fibroblasts, thereby contributing to cardiac fibrosis^12^. Despite increasing interest, the specific role of m6A methylation in cardiac fibrosis and its underlying molecular mechanisms remain incompletely understood.

Transverse Aortic Constriction (TAC) is a well-established experimental model for heart failure, characterized by the induction of cardiac hypertrophy and fibrosis due to mechanical pressure overload, ultimately leading to heart failure [13]. This study aimed to investigate the dynamic changes in m6A methylation within the TAC-induced cardiac fibrosis model and to elucidate its regulatory mechanisms on cardiac function. Using MeRIP-seq technology, we performed a comprehensive analysis of the global m6A methylation patterns in myocardial tissue from the TAC model. Furthermore, we assessed the expression changes of m6A methylation-modifying enzymes and their potential impact on the progression of cardiac fibrosis.

## Materials and methods

### Animals

Twenty male C57BL/6 mice (SPF grade, 7–8 weeks old) were purchased from the Lanzhou Veterinary Research Institute, Chinese Academy of Agricultural Sciences (Lanzhou, China, license No. 2020-0002). The mice were housed in a controlled environment at 24 ± 1 °C, with a relative humidity of 55 ± 5%, and a 12-hour light/dark cycle. They had ad libitum access to food and water. All procedures were conducted in compliance with relevant guidelines and regulations and are reported following the ARRIVE guidelines (https://arriveguidelines.org/) for animal experiments.

### Grouping of mice and establishment of the transverse aortic constriction (TAC) model

Mice were randomly allocated into two groups, each consisting of 6 to 10 mice. Over a period of seven days, the mice were subjected to a controlled feeding regimen. An anesthesia machine was utilized to administer anesthesia to the small animals by mixing pure oxygen with volatilized isoflurane. Anesthesia induction was achieved with an isoflurane concentration of 2–3% over 2–3 min, followed by maintenance of anesthesia at a concentration of 1.5-2%. In the model group, the mice were placed in a supine position on a surgical platform. A midline incision, approximately 10 mm long, was made from the mid-neck to the sternum to allow access to the thoracic cavity. The skin and underlying tissues were carefully dissected to expose the trachea and thoracic inlet. The pretracheal muscles were separated to provide access to the aortic arch. Using micro-surgical forceps and scissors, the mediastinal fat and thymic tissue were gently retracted to visualize the aortic arch. A 27-gauge blunt-end needle was inserted between the right subclavian artery and the left common carotid artery. A 6 − 0 polypropylene suture was utilized to ligate the aorta around the needle, ensuring a narrowing of approximately 0.4 mm in diameter. Once the ligation was confirmed, the needle was removed, and the thoracic cavity was closed layer by layer, starting with the muscles and ending with the skin. The mice were allowed to recover on a heated pad before being returned to their cages, where they had ad libitum access to food and water. For the sham group, mice underwent identical surgical procedures up to the point of ligation. Instead of ligating the aorta, the aortic arch was only exposed and visualized without any constriction applied. The thoracic cavity was then closed in the same manner as the model group.

### Echocardiographic assessment

Four weeks after surgery, echocardiographic evaluations were performed by technicians who were unaware of the experimental groups. Mice were anesthetized with an inhalational anesthetic, and their heart rates were monitored and maintained between 400 and 500 beats per minute. The mice were placed in a supine position on a heating pad set at 37 °C to prevent hypothermia. The anterior chest wall was shaved and cleaned to improve ultrasound imaging clarity. Standard views including the left ventricular long-axis, short-axis, and aortic arch views were captured using a high-frequency ultrasound system (Mylab X5 Vet, BersinBio, Guangzhou, China) designed for small animal imaging. Key measurements included: Left ventricular (LV) ejection fraction (LVEF), LV end-diastolic diameter (LVIDd), LV end-systolic diameter (LVIDs), LV posterior wall thickness, LV posterior wall thickness (LVPWd) (end-diastolic), and LV posterior wall thickness (LVPWs) (end-systolic).

### Masson staining

To prepare cardiac tissue sections for Masson staining, a detailed protocol was followed: Paraffin-embedded sections were first deparaffinized in xylene for 30 min, followed by sequential dehydration using ethanol solutions. Each step in the dehydration process lasted 5 min, concluding with a rinse in distilled water for 2 min. The sections were then immersed in potassium dichromate overnight and subjected to heat treatment at 63 °C for 1 h. Afterward, the sections were stained with ponceau fuchsin solution for 10 min and gently rinsed with distilled water. Collagen fibers were then treated with phosphomolybdic acid until the color diminished. Aniline blue staining was applied for approximately 2 min. Dehydration was achieved using a graded alcohol series, followed by clearing with a transparency agent and mounting with neutral gum. Microscopic images were acquired using a micrographic imaging system.

### Hematoxylin and Eosin (HE) staining

Cardiac tissues were harvested, fixed in 4% paraformaldehyde overnight, processed, and subsequently embedded in paraffin. Following this, the tissue sections were stained with HE to assess the extent of lesion formation and inflammatory cell infiltration under 400 x magnification using the Pannoramic MIDI II histological scanner (3DHistech; Budapest, Hungary).

### Sirius red staining

Myocardial tissue wax blocks were sliced into 4 μm sections using a paraffin microtome. The slices were dewaxed and hydrated by soaking in xylene I and II for 25 min each, followed by ethanol of varying gradients for 10 min each. After rinsing with tap water and PBS, iron hematoxylin dye (mixed 1:1) was applied for 5–10 min and rinsed off with distilled water. Sirius red dye was then applied for 15–30 min, followed by a rinse with running water. The sections were dehydrated in 75% and 95% ethanol, cleared with xylene three times for 1–2 min each, and finally sealed with neutral gum. Pathological changes were observed using the Pannoramic MIDI II histological scanner (3DHistech; Budapest, Hungary).

### Western blot analysis

Total protein was extracted from 0.2 g of cardiac tissue using 200 µL of RIPA tissue lysate (Servicebio, Wuhan, China, #G2002). Following extraction, a 10% SDS-PAGE gel was prepared, and 30 µg of total protein was electrophoretically separated. The proteins were then transferred onto a polyvinylidene difluoride (PVDF) membrane (Millipore Corporation, USA, #IPVH00010) and blocked with 5% skim milk powder (Biosharp, Beijing, China, #BS102-500 g) at room temperature for 2 h. For antibody incubation, the primary antibody was diluted according to the manufacturer’s protocol and applied to the PVDF membrane in a prepared working solution. The antibodies used are listed in Table 1. The membrane was then incubated overnight at 4 °C on a shaker in a refrigerator. After the primary antibody incubation, the PVDF membrane was washed with Tris-buffered saline with Tween 20 (TBST, Solaibao, Beijing, China, #T8220). A horseradish peroxidase-conjugated goat secondary antibody was applied to the membrane and incubated for 2 h at room temperature on a shaker, followed by additional washes. Finally, the exposure analysis was conducted by developing the PVDF membrane using enhanced chemiluminescence (ECL, Biosharp Co., Ltd, Guangzhou, China, #BL520B).

**Table 1:** The protein primary antibodies used in Western blot analysis

Antibody	Dilution	Source	Article no.
METTL14	1/1000	Fine Test, Wuhan Fine Biotech Co., Wuhan, China	FNab10824
METTL3	1/1000	Fine Test, Wuhan Fine Biotech Co., Wuhan, China	FNab05139
ALKBH5	1/1000	Fine Test, Wuhan Fine Biotech Co., Wuhan, China	FNab00314
FTO	1/1000	Fine Test, Wuhan Fine Biotech Co., Wuhan, China	FNab09787
β-actin	1/5000	ImmunoWay, Plano, TX, USA	YT0099

### Dot blot analysis

Cardiac tissue was homogenized in TRIpure Total RNA Extraction Reagent (ELK Biotechnology, Hubei, China, #EP013) and processed to extract total RNA following standard protocols. The RNA was diluted in RNase-free water, denatured at 95 °C for 5 min, and chilled on ice. It was then applied to a methanol-activated PVDF membrane (Millipore Corporation, USA, IPVH00010) and fixed with UV light for 10 min. The membrane was washed with Wash Buffer, blocked with 5% non-fat milk in PBS-T for 1 h, and incubated with the primary antibody (m6A, Proteintech, Wuhan, China, #68055-1-Ig) overnight at 4 °C. The next day, the membrane was washed three times with 10 mL of Wash Buffer for 5 min each at room temperature, then incubated with a diluted secondary antibody in 10 mL of blocking buffer for 1 h. Afterward, it was washed four times with 10 mL of Wash Buffer for 5 min each. The membrane was exposed to ECL substrate (Aspen Technology, Burlington, USA, AS1059) and visualized using chemiluminescence imaging.

### Real-time polymerase chain reaction (PCR)

The total RNA was extracted using the Total RNA Kit I (Omega Bio-Tek, Georgia, USA, #R6834-01) following the manufacturer’s instructions. The concentration and purity of the RNA solution were determined using UV spectrophotometry. One microliter of total RNA was reverse transcribed into complementary DNA (cDNA) using an Evo M-MLV RT Premix kit (Accurate Biology, China, #AG11728) for subsequent amplification as needed. PCR assays were conducted in a 25-microliter reaction volume, utilizing the β-actin gene as an internal control. The cycle threshold (Ct) values obtained from the PCR instrument were employed for the relative quantification of the initial templates. The primer sequences are shown in Table 2.

**Table 2:** Primer sequence of real-time PCR

Genes	Primer sequence 5’–3’	Amplified fragments/bp
β-actin	CATCCGTAAAGACCTCTATGCCAAC	171
	ATGGAGCCACCGATCCACA	
METTL3	GGACTCTGGGCACTTGGATTTA	248
	CAGGTGCATCTGGCGTAGAG	
METTL14	GACTGGCATCACTGCGAATG	126
	AGGTCCAATCCTTCCCCAGA	
ALKBH5	CACGTTGACCCCATCCACAT	240
	CCTGAGAATGATGACCGCCC	
FTO	TCTGTGTGTTGGGTGTCCTTT	143
	AAAACGACAGCGGTGCTTAC	

### Methylated RNA Immunoprecipitation sequencing (MeRIP-Seq)

The MeRIP-seq technique was employed to identify genome-wide regions with m6A modifications in mouse cardiac tissue. Total RNA was extracted using the Total RNA Kit I (Omega Bio-Tek, Georgia, USA, #R6834-01) following the manufacturer’s protocol. To eliminate potential DNA contamination, the sample was treated with DNase I (NEB, Ipswich, USA, M0303L). The purity of the extracted RNA was assessed by measuring the A260/A280 ratio using the Nanodrop™ OneC spectrophotometer (Thermo Fisher Scientific, Massachusetts, USA). RNA integrity was verified with the LabChip GX Touch system (Revvity, Shanghai, China). Subsequently, the concentration of the qualified RNA was determined using the Qubit 3.0 fluorometer and the QubitTM RNA Broad Range Assay kit (Thermo Fisher Scientific, Massachusetts, USA, Q10210). Total RNA was enriched for polyadenylated RNA (polyA + RNA) following the instructions provided with the VAHTS mRNA capture beads (Vazyme, Jiangsu, Nanjing, China, #N401).

In the m6A methylated RNA immunoprecipitation (meRIP) assay, polyA + RNA was fragmented to approximately 100 nucleotides by incubation with 20 mM ZnCl2 at 95 °C for 5–10 min. A 10% aliquot of this fragmented RNA was reserved as the “Input” control, while the remaining 90% underwent m6A immunoprecipitation. The fragmented polyA + RNA was combined with a specific anti-N6-methyladenosine polyclonal antibody (Synaptic Systems, Gottingen, Germany, #202003) and RNasin ribonuclease inhibitor (Promega, Madison, WI, USA, #N2615) to achieve a final concentration of 40 U/µL, followed by incubation at 4 °C for 2 h. This antibody specifically targets m6A-modified RNA molecules. Subsequently, the RNA-antibody complexes were captured using protein G magnetic beads (Thermo Fisher Scientific, Massachusetts, USA, 88848), which were incubated at 4 °C for 1 h to ensure efficient binding. The beads were then thoroughly washed to remove non-specifically bound RNA, and the immunoprecipitated RNA was isolated using TRIzol reagents.

RNA sequencing libraries were constructed from input samples and immunoprecipitated RNA following the protocol outlined by the KC™ Digital mRNA Library Prep Kit (SEQHEALTH, Wuhan, China), using specific components provided in the kit. The library preparation process involved enriching PCR products within a size range of 200 to 500 base pairs. Subsequently, the libraries were quantified and sequenced using the DNBSEQ-T7 platform (MGI Tech Co, Shenzhen, China) with the PE150 sequencing model.

### Statistical analysis

Data are expressed as means ± standard deviation (SD). Statistical analyses were conducted utilizing SPSS software (version 19.0, SPSS Inc., Chicago, IL, USA). Kolmogorov-Smirnov tests confirmed that the data adhered to a normal distribution, meeting parametric assumptions. Comparisons between two independent groups were performed using Student’s T-test. Statistically significant differences were considered at *p* < 0.05.

## Results

### TAC induced impaired cardiac function and left ventricular remodeling in mice

**Fig. 1:**
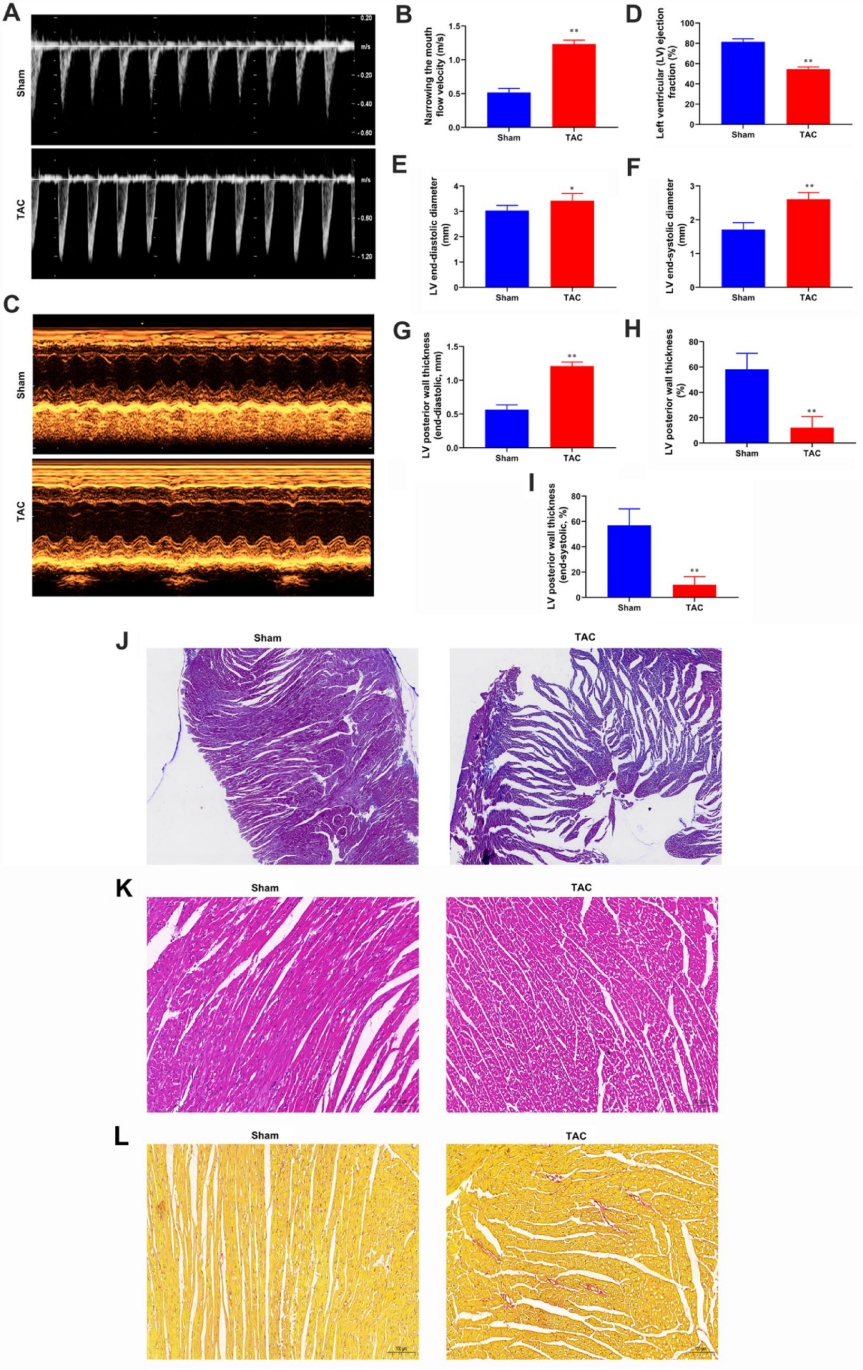
TAC induced impaired cardiac function and left ventricular remodeling in mice. **A**, **B** Echocardiographic assessment of aortic valve blood flow velocity in sham and TAC groups. **C**–**I** Cardiac function in mice was assessed using echocardiography. Key measurements included: Ejection fraction (EF), Left ventricular (LV) ejection fraction (LVEF), LV end-diastolic diameter (LVIDd), LV end-systolic diameter (LVIDs), LV posterior wall thickness, LV posterior wall thickness (LVPWd) (end-diastolic), LV posterior wall thickness (LVPWs) (end-systolic). **J** Masson staining was used to observe collagen deposition in the cardiac tissue of TAC-induced mice at a magnification of 400x. **K** HE staining showing pathological changes in the cardiac tissues. **L** Sirius red staining indicating fibrosis in the cardiac tissue of TAC-induced mice. ^**^P < 0.01 vs. sham. Data is presented as mean ± SD

Four weeks after the TAC operation, echocardiography was used to measure blood flow velocity in both the TAC group and the sham group (Fig. 1A and B). The elevated peak velocity in the left ventricular outflow tract serves as a critical parameter confirming successful pressure overload and effective establishment of the TAC model. This increase reflects the hemodynamic burden imposed by aortic constriction. The results indicated a significant increase in blood flow velocity in the TAC group, while the sham group’s blood flow velocity remained normal (Fig. 1A and B). These findings confirmed the successful establishment of a myocardial fibrosis model in mice after TAC surgery (*P* < 0.01, Fig. 1A and B). By quantifying left ventricular functional and structural parameters, we conducted a comprehensive evaluation of the impact of TAC surgery on murine cardiac function. The LVEF in the TAC group was significantly lower than in the sham group (*P* < 0.01), indicating that TAC surgery significantly impaired cardiac ejection performance (Fig. 1C and D). Additionally, the LVIDd, LVIDs, and LV posterior wall thickness in the TAC group were significantly greater than those in the sham group (*P* < 0.01, Fig. 1C, E and G). Furthermore, the LVPWd and LVPWs in the TAC group were significantly reduced compared to the sham group (*P* < 0.01, Fig. 1C, H and I). Masson staining revealed increased collagen deposition in the cardiac tissue of TAC-induced mice compared to the control group (Fig. 1J). These results indicated that TAC led to cardiac dysfunction and myocardial fibrosis. HE staining results demonstrated that, in comparison to the control group, the myocardium in the model group exhibited disorganization and vacuolization, with shortened myofiber lengths and enlarged interstitial spaces (Fig. 1K). Sirius red staining further revealed that the muscle fibers in the model group were significantly disordered, accompanied by marked fibrosis and a substantial increase in collagen fibers (Fig. 1L). These results indicated that TAC resulted in cardiac dysfunction and myocardial fibrosis.

### Enhanced m6A methylation and dysregulation of m6A modifying enzymes in myocardial tissues of TAC-induced cardiac fibrosis model

**Fig. 2:**
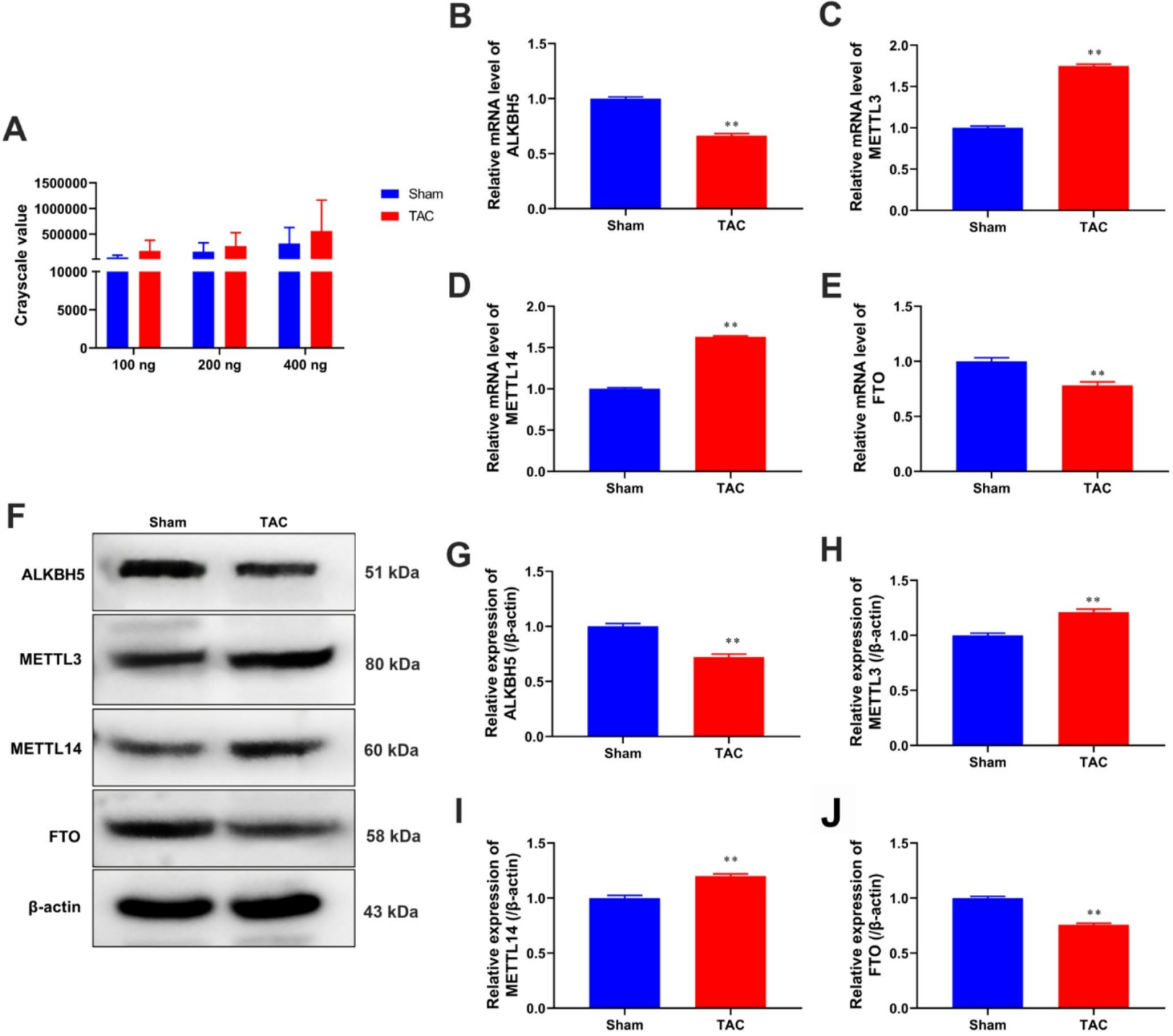
Shows enhanced m6A methylation and dysregulation of m6A modifying enzymes in myocardial tissues of TAC-induced cardiac fibrosis model. **A** Dot blot analysis demonstrates increased global m6A methylation levels in myocardial tis-sues of the TAC group compared to the sham group (*P* > 0.05). (**B–E**) PCR analysis was used to test mRNA levels of key m6A methyltransferases (METTL3, METTL14) and demethylases (ALKBH5, FTO) in myocardial tis-sues from the TAC and sham groups. **F–J** Western blot analysis was performed on ALKBH5, METTL3, METTL14, and FTO in myocardial tissues. β-actin was used as internal control. ^*^P < 0.05, ^**^P < 0.01 vs. sham groups. Data is presented as mean ± SD

Next, we utilized dot blot analysis to assess the global m6A methylation status in myocardial tissues from both TAC and sham groups at 4 weeks post-surgery (*P* < 0.05, Fig. 2A). The results demonstrated a significant increase in m6A methylation levels in the TAC group compared to the sham group, suggesting that m6A methylation is dynamically regulated and may contribute to the progression of cardiac fibrosis (Fig. 2A). To further understand the mechanisms behind the observed changes in m6A methylation, we measured the expression levels of key m6A methylation modifying enzymes in myocardial tissues from both TAC and sham groups. Through PCR and Western blot analyses, we assessed the expression of m6A methyltransferases (METTL3 and METTL14) and demethylases (ALKBH5 and FTO). Our findings revealed that the mRNA and protein expression levels of METTL3 and METTL14 were significantly higher in the TAC group compared to the sham group, aligning with the observed increase in m6A methylation levels (*P* < 0.05 and *P* < 0.01, Fig. 2B and G). In contrast, the expression levels of ALKBH5 and FTO were notably lower in the TAC group, providing further that m6A methylation was intricately regulated by these enzymes during cardiac fibrosis (*P* < 0.05 and *P* < 0.01, Fig. 2B and G).

### General characteristics of m6A methylation modification in cardiac tissue in TAC-induced cardiac fibrosis model

**Fig. 3:**
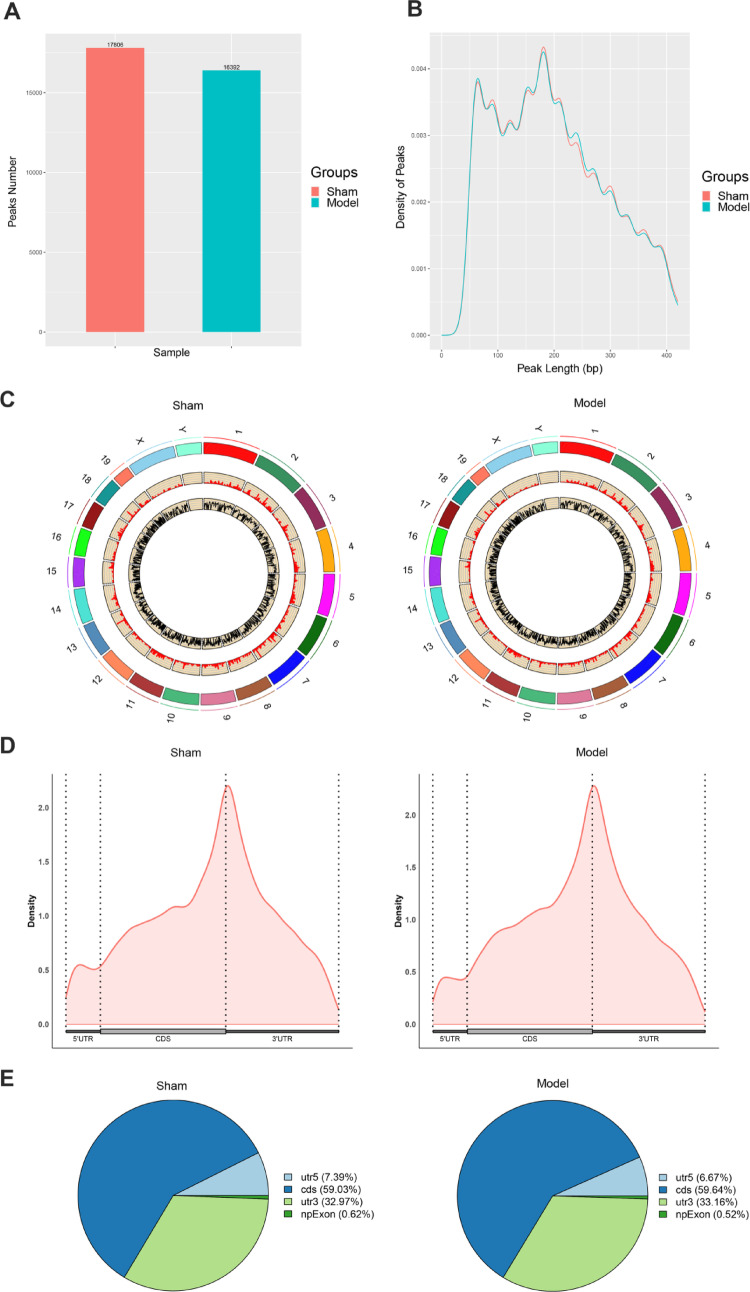
General characteristics and topological characteristics of m6A methylation modification in cardiac tissue in TAC-induced cardiac fibrosis model. (**A** and **B**) Statistics on peak number and length statistics. (**C**) Distribution of peaks in chromosomes. (**D**) Distribution of peaks in the 5’ untranslated region (5’UTR), coding sequence (CDS), 3’UTR of genes. (**E**) Statistical distribution of peaks in each functional region of the gene

Statistical analysis was conducted on the peak number and length of m6A-modified genes in both groups. The group that underwent a sham operation exhibited 17,806 peaks, while the TAC model group had 16,392 peaks (Fig. 3A). There was no significant difference observed in the distribution proportion of peaks between the two groups (Fig. 3B). The m6A locus map revealed a uniform distribution of m6A peaks across all chromosomes in both sample groups (Fig. 3C).

### Topological characteristics of m6A methylation modification in cardiac tissue in TAC-induced cardiac fibrosis model

To investigate the topological structure of m6A modifications in myocardial tissue following TAC induction, we analyzed the distribution of m6A peaks across transcripts. Based on the distribution of m6A sites, RNA transcripts were categorized into four distinct regions: the 3’ untranslated region (3’UTR), coding sequence (CDS), the npExon, and the 5’-UTR. The results demonstrated that the highest proportion of m6A peaks was enriched in the 3’ UTR region in both the sham operation group and the model group (Fig. 3D and E).

### Effect of TAC induction on m6A modification in mouse myocardial tissue

**Fig. 4:**
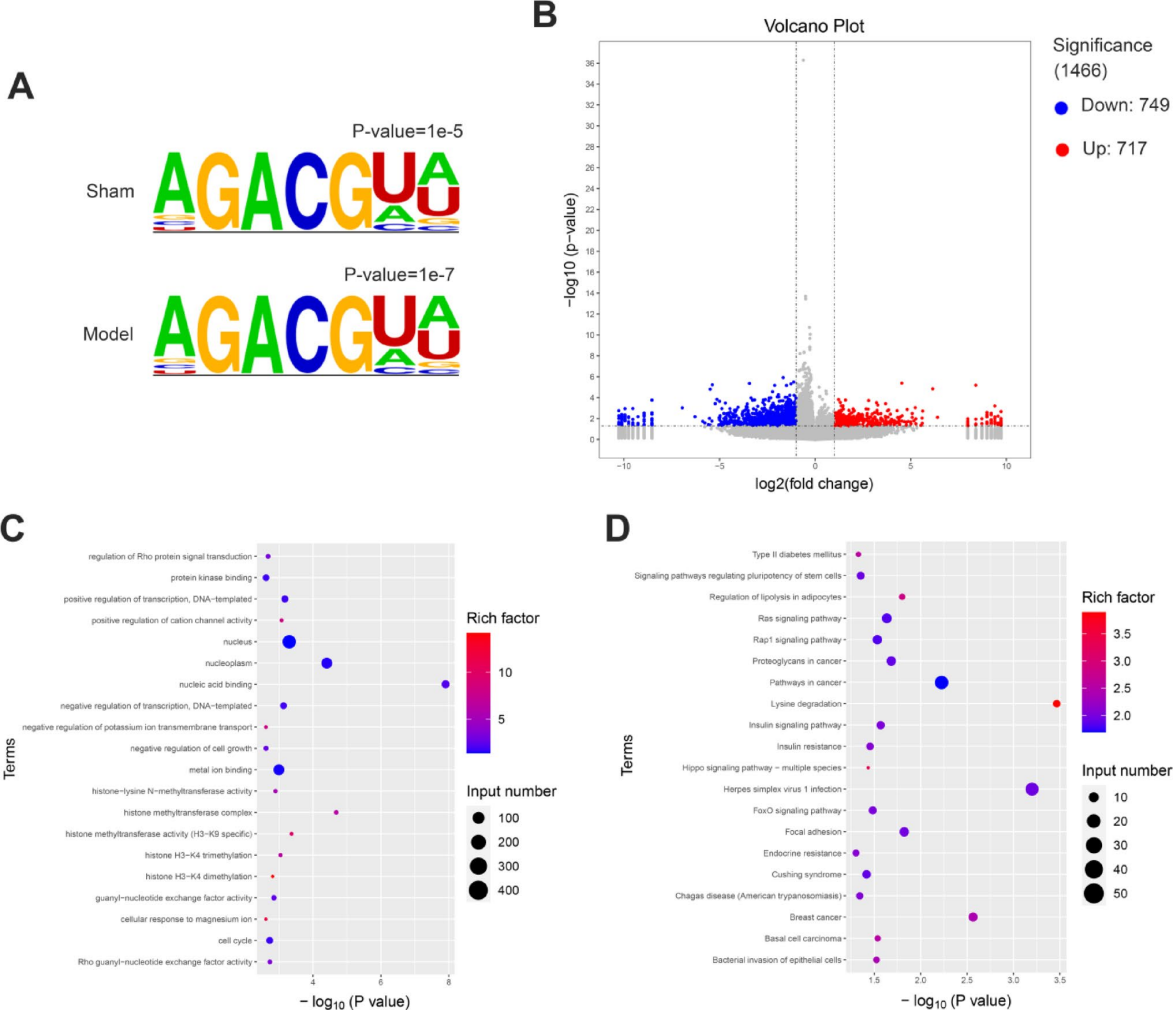
Effect of TAC induction on m6A modification in mouse myocardial tissue. **A** Homer known motif enrichment results. **B** Difference peak volcano map. **C** The Gene Ontology (GO) pathway enrichment map of differentially peak related genes. **D** Kyoto Encyclopedia of Genes and Genomes (KEGG) pathway enriched in genes related to peaks

The motifs in the samples from both groups were analyzed using HOMER software. The results indicated that motifs corresponding to the canonical m6A consensus sequence (RRACH, R = A/G and H = A/C/U) were identified in both the sham and model groups (Fig. 4A). As shown in Fig. 4B, a total of 1466 distinct m6A modification peaks were identified, with |log2FC| ≥ 0.58496 and *P* < 0.05. These peaks comprised 717 hypermethylated sites and 749 hypomethylated sites (Fig. 4B).

### TAC induced functional enrichment of abnormal m6A modified genes

To further investigate the functional roles of genes with aberrant m6A modifications in myocardial tissues, we performed Gene Ontology (GO) and Kyoto Encyclopedia of Genes and Genomes (KEGG) pathway enrichment analyses on the Differentially Methylated Genes (DMGs) identified between the two groups. The GO analysis revealed that DMGs were significantly enriched in Regulation of Rho protein signal transduction, Protein kinase binding, Positive regulation of transcription, DNA-templated, positive regulation of cation channel activity, Nucleus, Nucleoplasm, Nucleic acid binding, negative regulation of transcription, DNA-templated, Negative regulation of potassium ion transmembrane transport, Negative regulation of cell growth, and more (Fig. 4C). Importantly, the KEGG pathway analysis highlighted the involvement of DMGs in key signaling pathways, such as Type ll diabetes mellitus, signaling pathways regulating pluripotency of stem cells, Regulation of lipolysis in adipocytes, ras signaling pathway, Rap1 signaling pathway, proteoglycans in cancer, pathways in cancer, lysine degradation, insulin signaling pathway, and insulin resistance (Fig. 4D). These findings emphasize the potential regulatory roles of m6A modifications in myocardial tissues.

## Discussion

The TAC procedure is a well-established method used to induce pressure overload and subsequent cardiac fibrosis in mice [13, 14]. In our study, four weeks after TAC, echocardiographic analysis revealed significant impairments in cardiac function and left ventricular remodeling. The echocardiographic parameters, including EF, FS, LVIDd, and LVIDs, were markedly altered in the TAC group compared to the sham group. These findings are consistent with previous reports that have shown TAC-induced pressure overload leading to myocardial hypertrophy, fibrosis, and reduced cardiac contractility [15, 16]. Furthermore, the LVPWd and LVPWs were significantly reduced in the TAC group, indicating mild myocardial hypertrophy. This suggests that the initial stages of TAC-induced cardiac fibrosis may involve a complex interplay of cellular and molecular mechanisms, that require further elucidation.

Our study employed Dot blot analysis to evaluate the global m6A methylation status in myocardial tissues from both TAC and sham groups. The results demonstrated a significant increase in m6A methylation levels in the TAC group, suggesting that m6A methylation is dynamically regulated and may play a crucial role in the progression of cardiac fibrosis. This observation is consistent with previous studies that have demonstrated altered m6A methylation patterns in various cardiovascular diseases [17, 18]. To understand the mechanisms behind these changes, we measured the expression levels of key m6A methylation modifying enzymes, including methyltransferases (METTL3 and METTL14) and demethylases (ALKBH5 and FTO). Our findings indicated that the mRNA and protein expression levels of METTL3 and METTL14 were significantly higher in the TAC group, while the expression of ALKBH5 and FTO were significantly lower. These results align with the observed increase in m6A methylation levels. The METTL3-METTL14 complex acts as a methyltransferase, where METTL3 functions as the catalytic core and METTL14 acts as an RNA-binding platform, facilitating the transfer of methyl groups to target adenosine residues [19]. This methylation process is tightly regulated and reversible, with demethylases such as ALKBH5 and FTO responsible for removing these methyl groups, thus modulating the m6A landscape and affecting gene expression dynamics [20]. The upregulation of METTL3 and METTL14, along with the downregulation of ALKBH5 and FTO, suggests an imbalance between m6A methylation and demethylation in TAC-induced cardiac fibrosis. This dysregulation may contribute to changes in the expression of downstream target genes, potentially impacting cardiac function and left ventricular remodeling.

To gain a deeper understanding of the topological structure of m6A modifications in myocardial tissue following TAC induction, we conducted an analysis of the distribution of m6A peaks across transcripts. Our results revealed that the highest proportion of m6A peaks was enriched in the 3’UTR in both the Sham and TAC groups. This observation aligns with previous studies demonstrating that m6A peaks are predominantly located in the 3’UTR, where they play critical roles in regulating mRNA stability, splicing, and translation [21–23]. The enrichment of m6A peaks in the 3’UTR may have significant implications for gene expression regulation in TAC-induced cardiac fibrosis. For example, m6A modifications in the 3’UTR can influence the binding of RNA-binding proteins (RBPs) and microRNAs (miRNAs), thereby modulating mRNA stability and translation [24–26]. Consequently, dysregulation of m6A methylation in the 3’UTR may contribute to the aberrant expression of genes involved in cardiac function and remodeling.

The GO analysis indicated that DMGs were significantly enriched in several critical biological processes and molecular functions, such as the regulation of Rho protein signal transduction, protein kinase binding, and positive regulation of transcription. These processes are essential for cellular signaling, cytoskeletal organization, and gene expression, all of which are crucial for maintaining cardiac function and preventing pathological remodeling [27–29]. The KEGG pathway analysis further highlighted the involvement of DMGs in key signaling pathways, including the Ras, Rap1, and insulin signaling pathways. These pathways regulate cell growth, survival, and metabolism, and their dysregulation has been implicated in heart failure pathogenesis [30–33]. For instance, the Ras signaling pathway activates downstream effectors like MAPK and ERK, promoting myocardial hypertrophy [34, 35] and fibrosis [36]. Similarly, the Rap1 signaling pathway regulates cell adhesion and migration [37, 38], which are vital for maintaining cardiac structural integrity [39]. Moreover, the enrichment of DMGs in pathways related to type 2 diabetes mellitus and insulin resistance suggested that metabolic dysregulation may contribute to heart failure [40]. Metabolic disorders, such as insulin resistance and hyperglycemia, are established risk factors for cardiovascular diseases and can exacerbate heart failure progression [41]. Dysregulation of m6A methylation in these pathways may further intensify metabolic dysfunction, leading to a vicious cycle of cardiac injury and dysfunction.

### Limitations

Our study provides significant insights into the role of m6A methylation in TAC-induced cardiac fibrosis. However, several limitations must be acknowledged. Firstly, the research was conducted using a murine model, which may limit the direct applicability of the findings to human cardiac fibrosis. Future investigations should aim to validate these results in human samples to establish clinical relevance and explore the potential therapeutic implications of m6A methylation in cardiac fibrosis. Secondly, the study did not include functional validation of m6A-modified genes or their downstream targets. Additional experiments, such as gene knockdown or overexpression studies, are essential to confirm the causal relationship between m6A methylation and the progression of cardiac fibrosis. Thirdly, while the study assessed global changes in m6A methylation, it did not explore the specific mechanisms by which m6A modifications regulate gene expression. Although the enrichment of m6A modifications in 3’UTRs suggests possible interactions with RNA-binding proteins and miRNAs, this study did not experimentally assess these specific regulatory mechanisms. Future investigations using RNA immunoprecipitation assays and miRNA–mRNA interaction studies are warranted to elucidate how m6A methylation modulates transcript stability and translation through these mediators. Future research should utilize advanced techniques, such as CRISPR-based approaches, to identify precise m6A modification sites and elucidate their functional consequences.

## Conclusion

In conclusion, our research has revealed that m6A methylation is subject to dynamic regulation in cardiac fibrosis induced by TAC. This regulation contributes to the deterioration of cardiac function and remodeling of the left ventricle. The observed dysregulation of m6A methylation-modifying enzymes, along with the enrichment of m6A-modified genes within critical signaling pathways, underscores the pivotal role of m6A methylation in the pathogenesis of cardiac fibrosis. These findings provide a foundation for further investigation into the mechanisms by which m6A methylation influences cardiac fibrosis [42].

## Supplementary Information

Below is the link to the electronic supplementary material.

Supplementary Material 1

## Data Availability

The raw seguence data reported in this paper have been deposited in the Genome Se-quence Archive (GSA: CRA023973) that are publicly accessible at https:/ngdc.cncb.ac.cn/gsa42.
