# Supplementary figures and images for: Role of N6-methyladenosine methylation in transverse aortic constriction-induced cardiac fibrosis: insights from MeRIP-seq analysis
Source: Mol Biol Rep. 2025 Aug 26;52(1):850. doi: 10.1007/s11033-025-10940-2 (PMC12380991; doi:10.1007/s11033-025-10940-2)

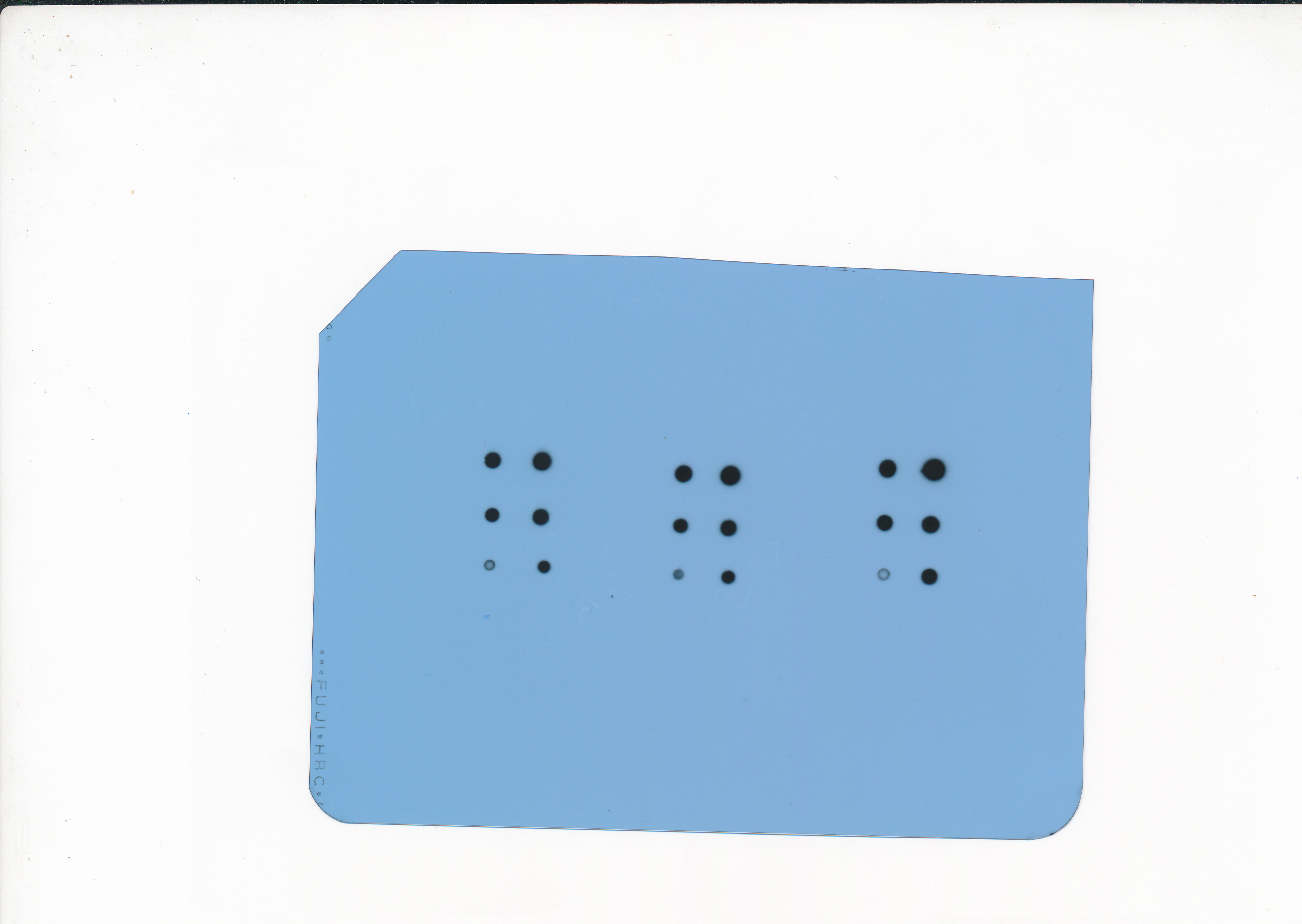

Supplement: Supplementary file 1 — Supplementary Material 1 [file 11033_2025_10940_MOESM1_ESM.zip › original versions/ dot blot σÄƒσ¢╛/σÄƒσ¢╛-2.tif]

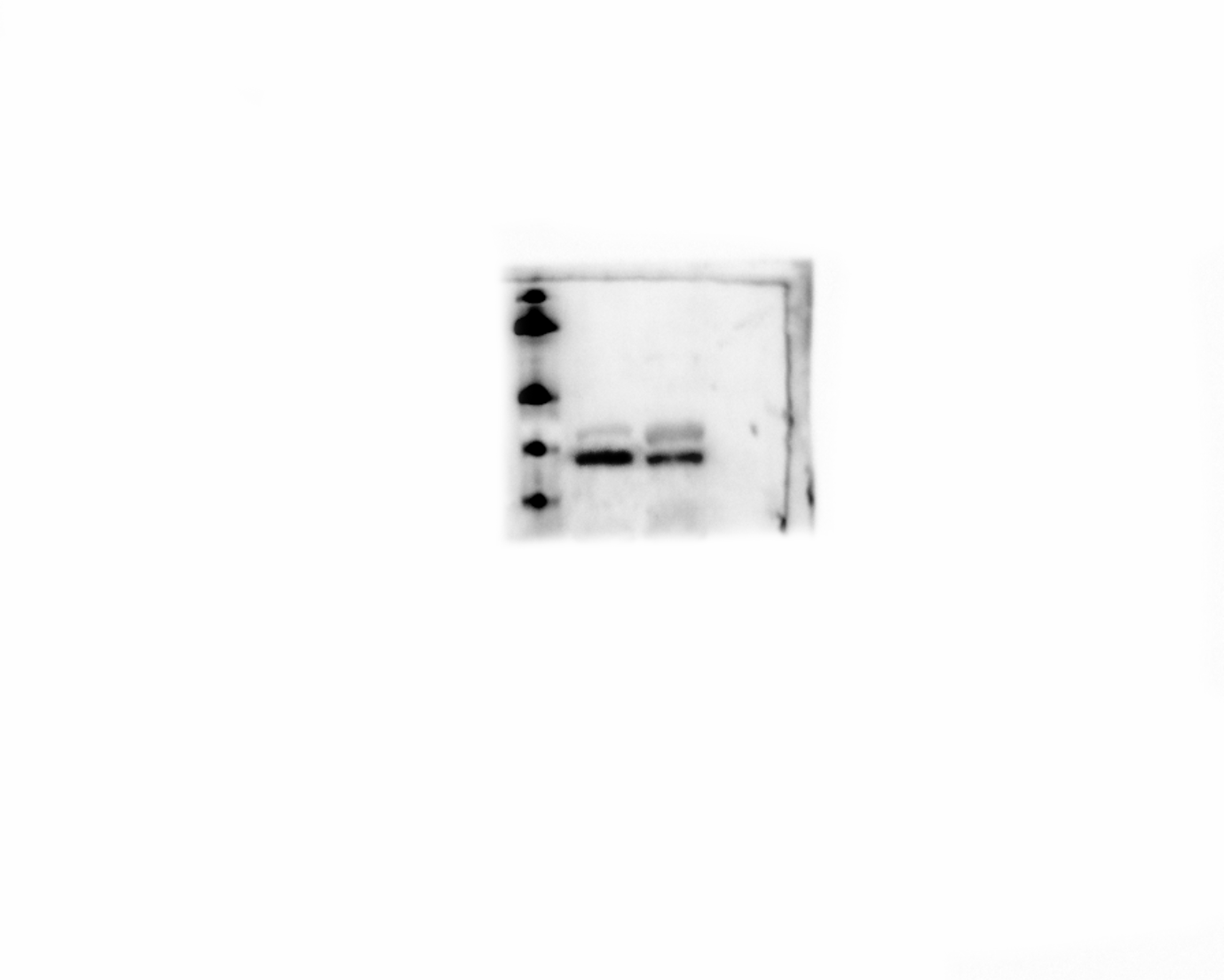

Supplement: Supplementary file 1 — Supplementary Material 1 [file 11033_2025_10940_MOESM1_ESM.zip › original versions/ΘçìσñìΣ╕ë/σÄƒσ¢╛/FTO.png]

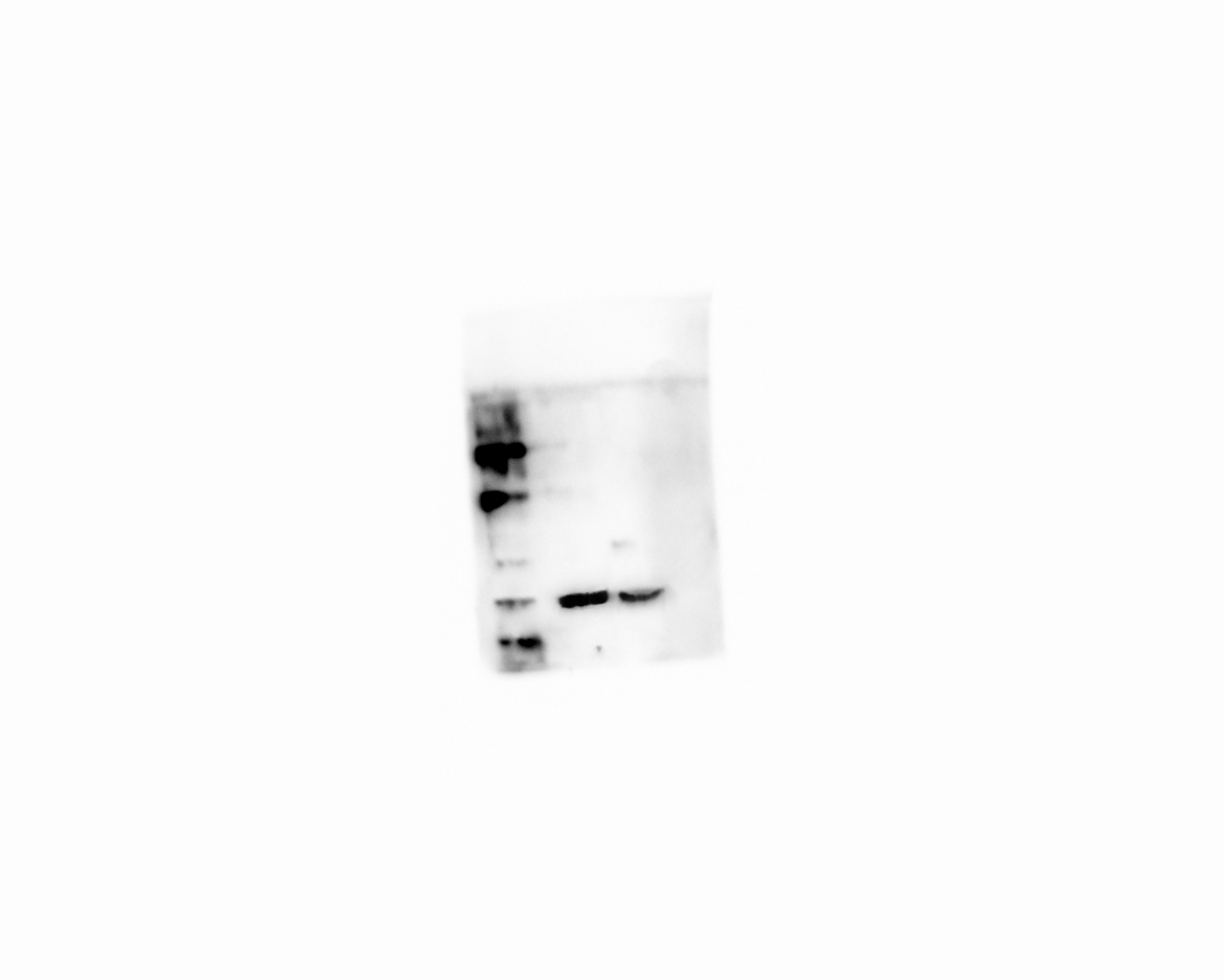

Supplement: Supplementary file 1 — Supplementary Material 1 [file 11033_2025_10940_MOESM1_ESM.zip › original versions/ΘçìσñìΣ╕ë/σÄƒσ¢╛/ALKBH5.png]

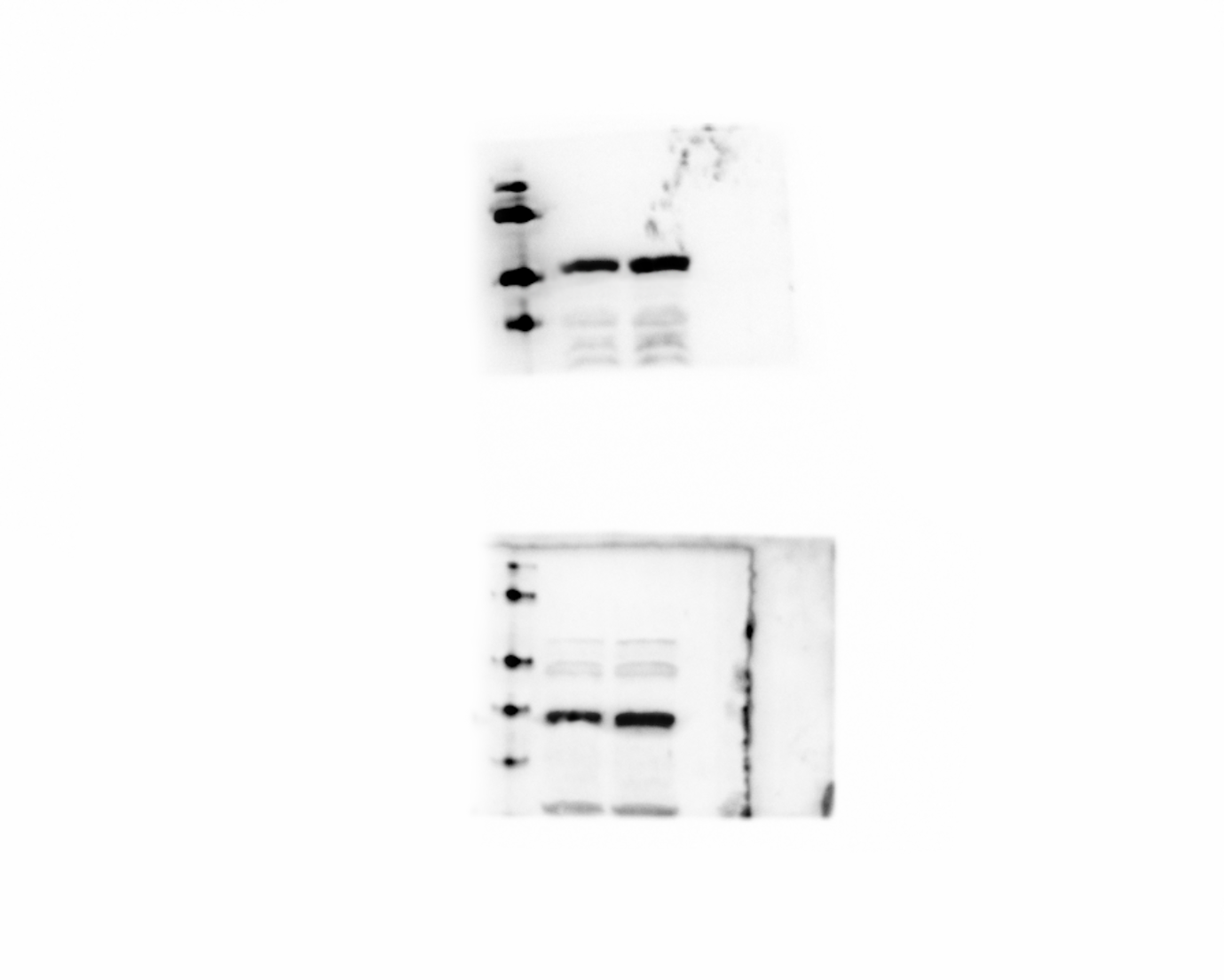

Supplement: Supplementary file 1 — Supplementary Material 1 [file 11033_2025_10940_MOESM1_ESM.zip › original versions/ΘçìσñìΣ╕ë/σÄƒσ¢╛/METTL3 METTL14.png]

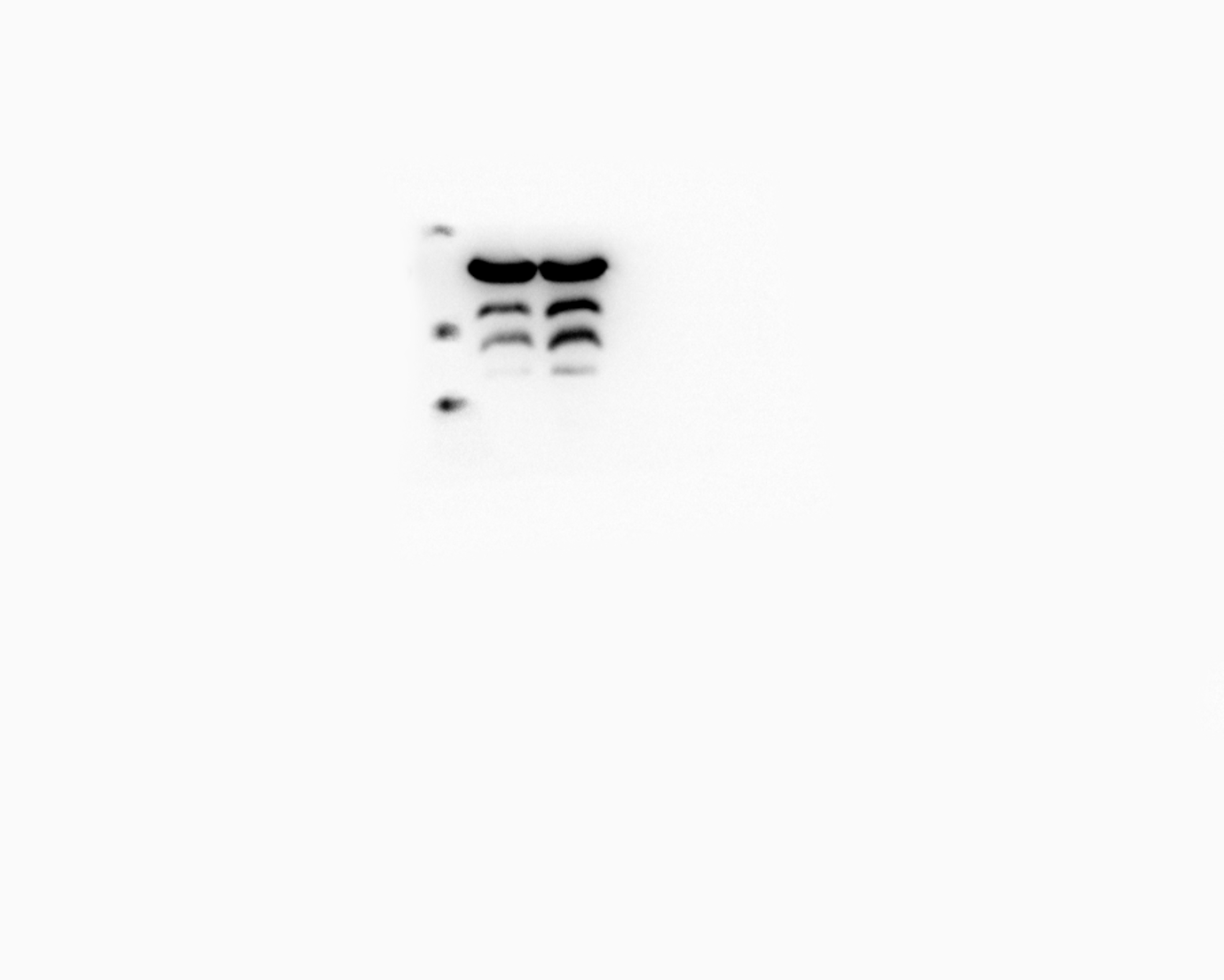

Supplement: Supplementary file 1 — Supplementary Material 1 [file 11033_2025_10940_MOESM1_ESM.zip › original versions/ΘçìσñìΣ╕ë/σÄƒσ¢╛/╬▓-actin-1.png]

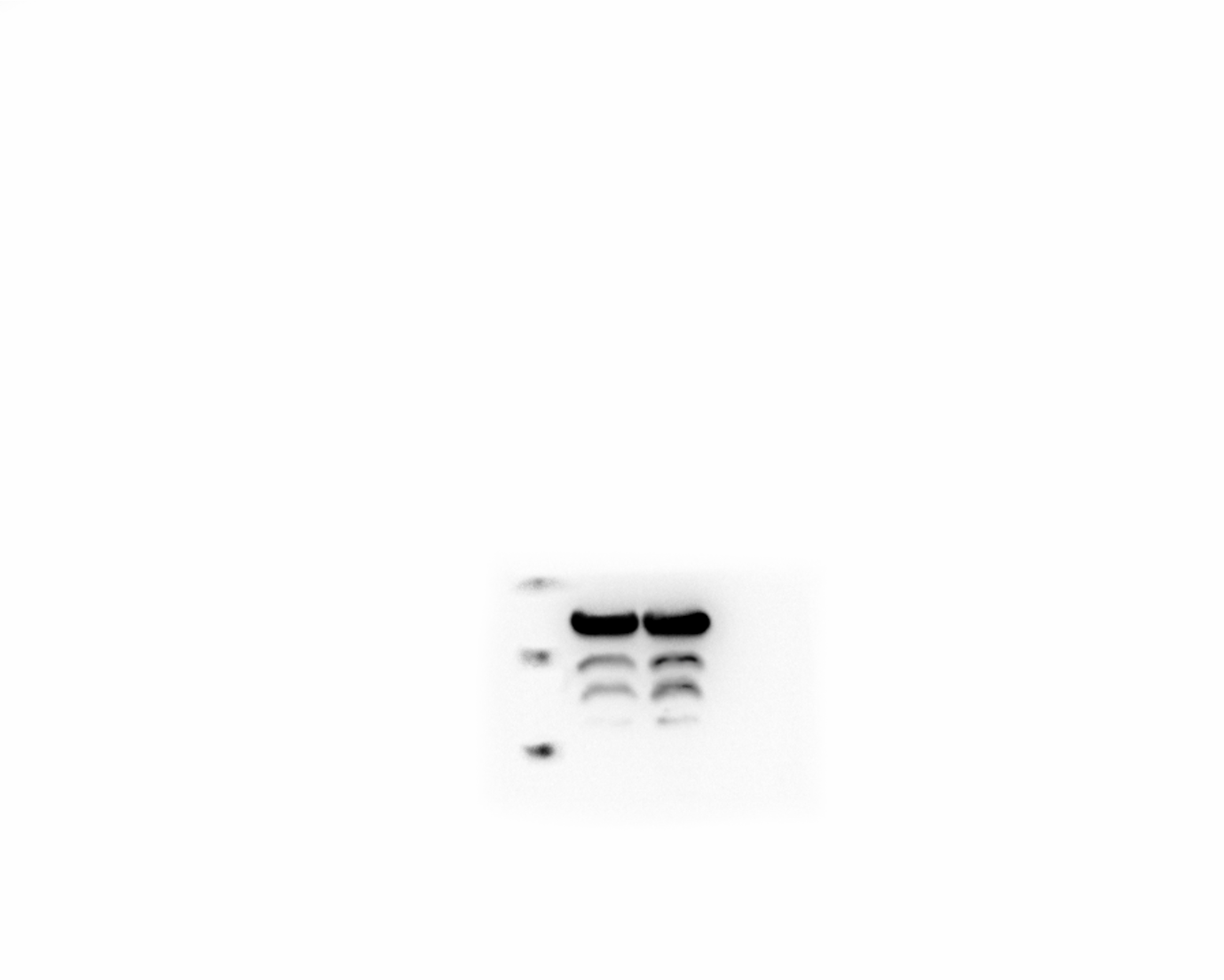

Supplement: Supplementary file 1 — Supplementary Material 1 [file 11033_2025_10940_MOESM1_ESM.zip › original versions/ΘçìσñìΣ╕ë/σÄƒσ¢╛/╬▓-actin.png]

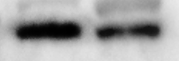

Supplement: Supplementary file 1 — Supplementary Material 1 [file 11033_2025_10940_MOESM1_ESM.zip › original versions/ΘçìσñìΣ╕ë/μ¥íσ╕a/FTO.png]

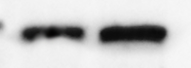

Supplement: Supplementary file 1 — Supplementary Material 1 [file 11033_2025_10940_MOESM1_ESM.zip › original versions/ΘçìσñìΣ╕ë/μ¥íσ╕a/METTL14.png]

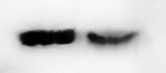

Supplement: Supplementary file 1 — Supplementary Material 1 [file 11033_2025_10940_MOESM1_ESM.zip › original versions/ΘçìσñìΣ╕ë/μ¥íσ╕a/ALKBH5.png]

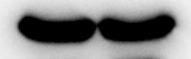

Supplement: Supplementary file 1 — Supplementary Material 1 [file 11033_2025_10940_MOESM1_ESM.zip › original versions/ΘçìσñìΣ╕ë/μ¥íσ╕a/╬▓-actin-1.png]

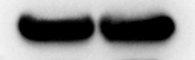

Supplement: Supplementary file 1 — Supplementary Material 1 [file 11033_2025_10940_MOESM1_ESM.zip › original versions/ΘçìσñìΣ╕ë/μ¥íσ╕a/╬▓-actin.png]

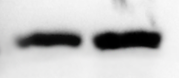

Supplement: Supplementary file 1 — Supplementary Material 1 [file 11033_2025_10940_MOESM1_ESM.zip › original versions/ΘçìσñìΣ╕ë/μ¥íσ╕a/METTL3.png]

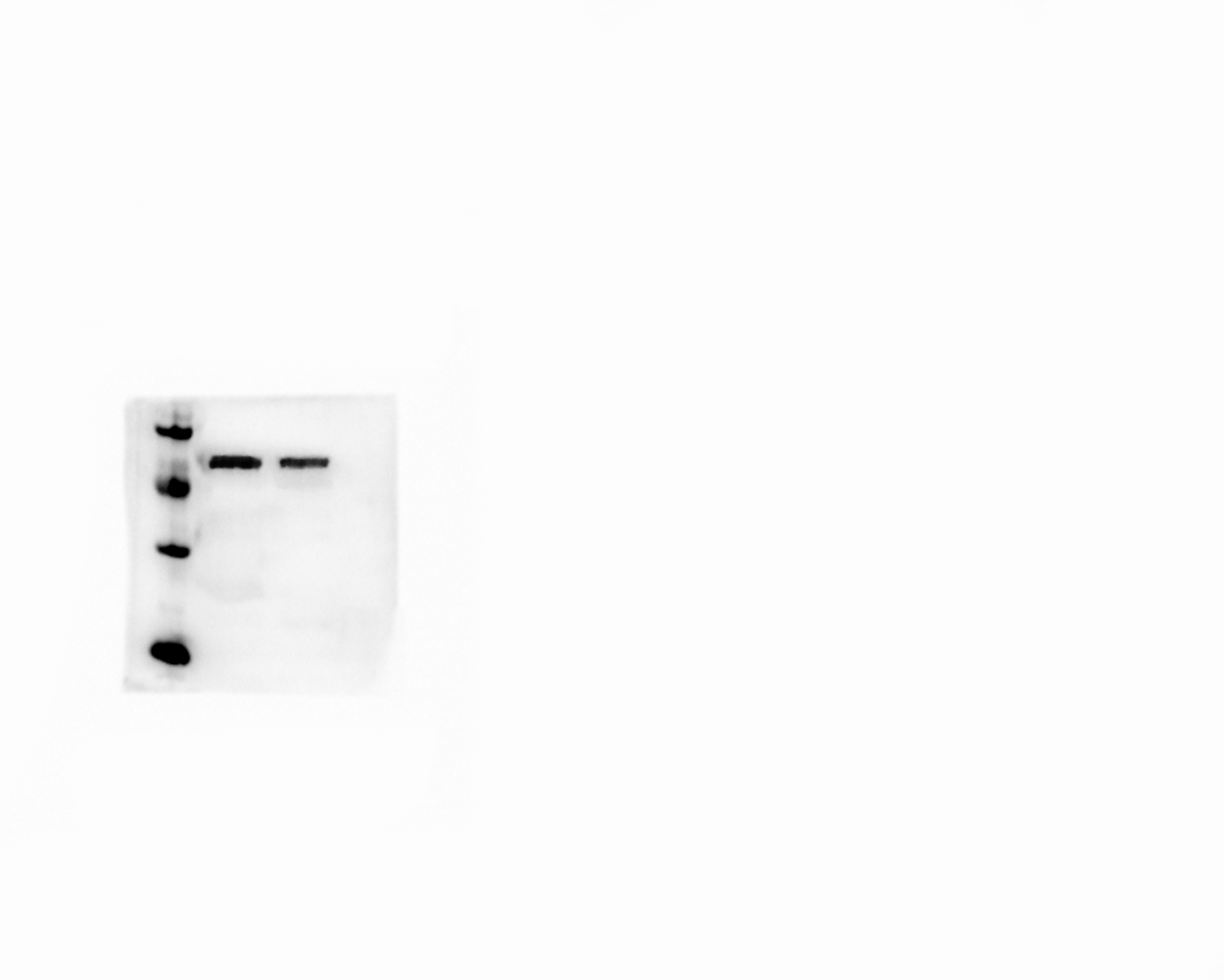

Supplement: Supplementary file 1 — Supplementary Material 1 [file 11033_2025_10940_MOESM1_ESM.zip › original versions/ΘçìσñìΣ╕Ç/σÄƒσ¢╛/ALKBH5.png]

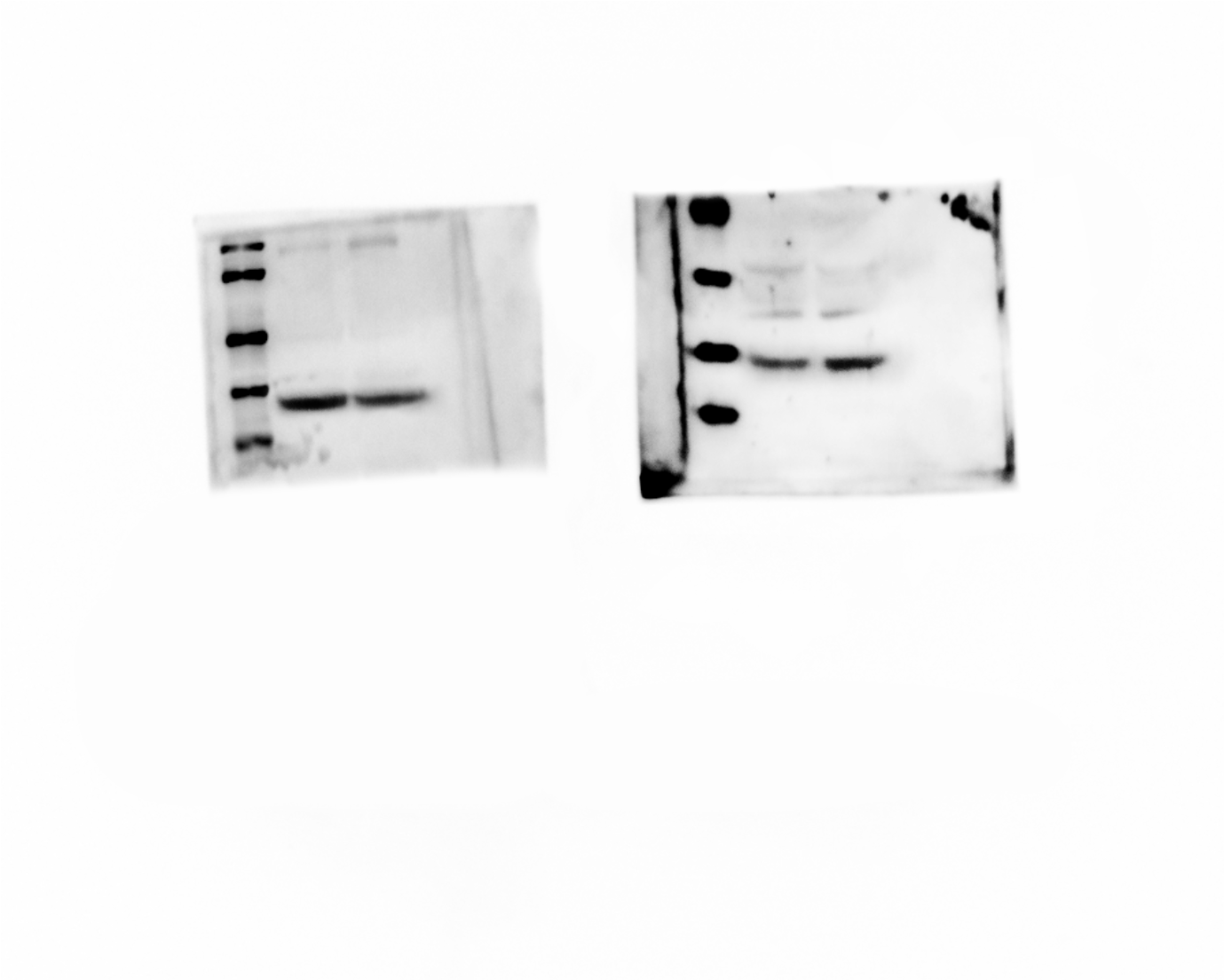

Supplement: Supplementary file 1 — Supplementary Material 1 [file 11033_2025_10940_MOESM1_ESM.zip › original versions/ΘçìσñìΣ╕Ç/σÄƒσ¢╛/FTO METTL14.png]

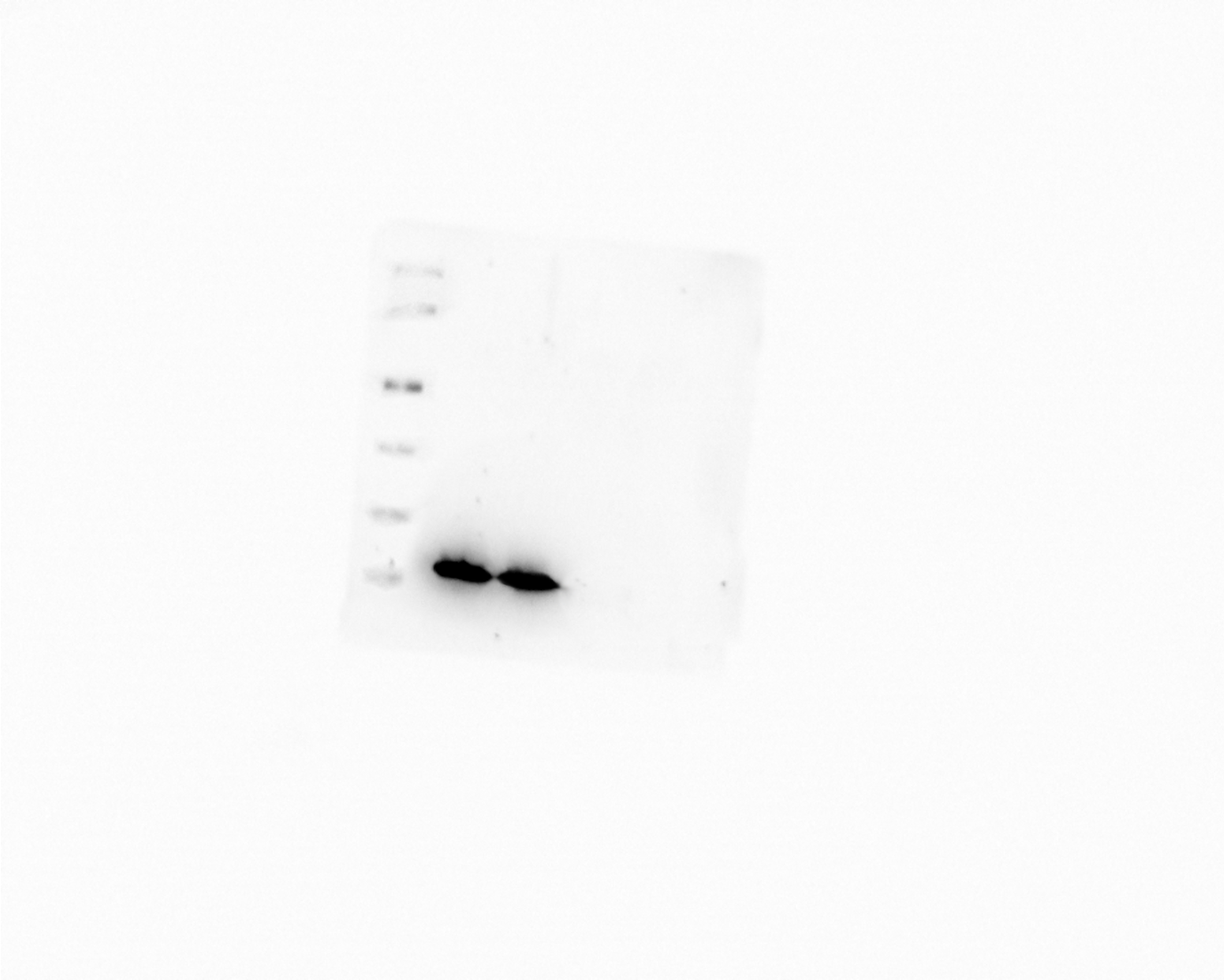

Supplement: Supplementary file 1 — Supplementary Material 1 [file 11033_2025_10940_MOESM1_ESM.zip › original versions/ΘçìσñìΣ╕Ç/σÄƒσ¢╛/╬▓-actin-1.png]

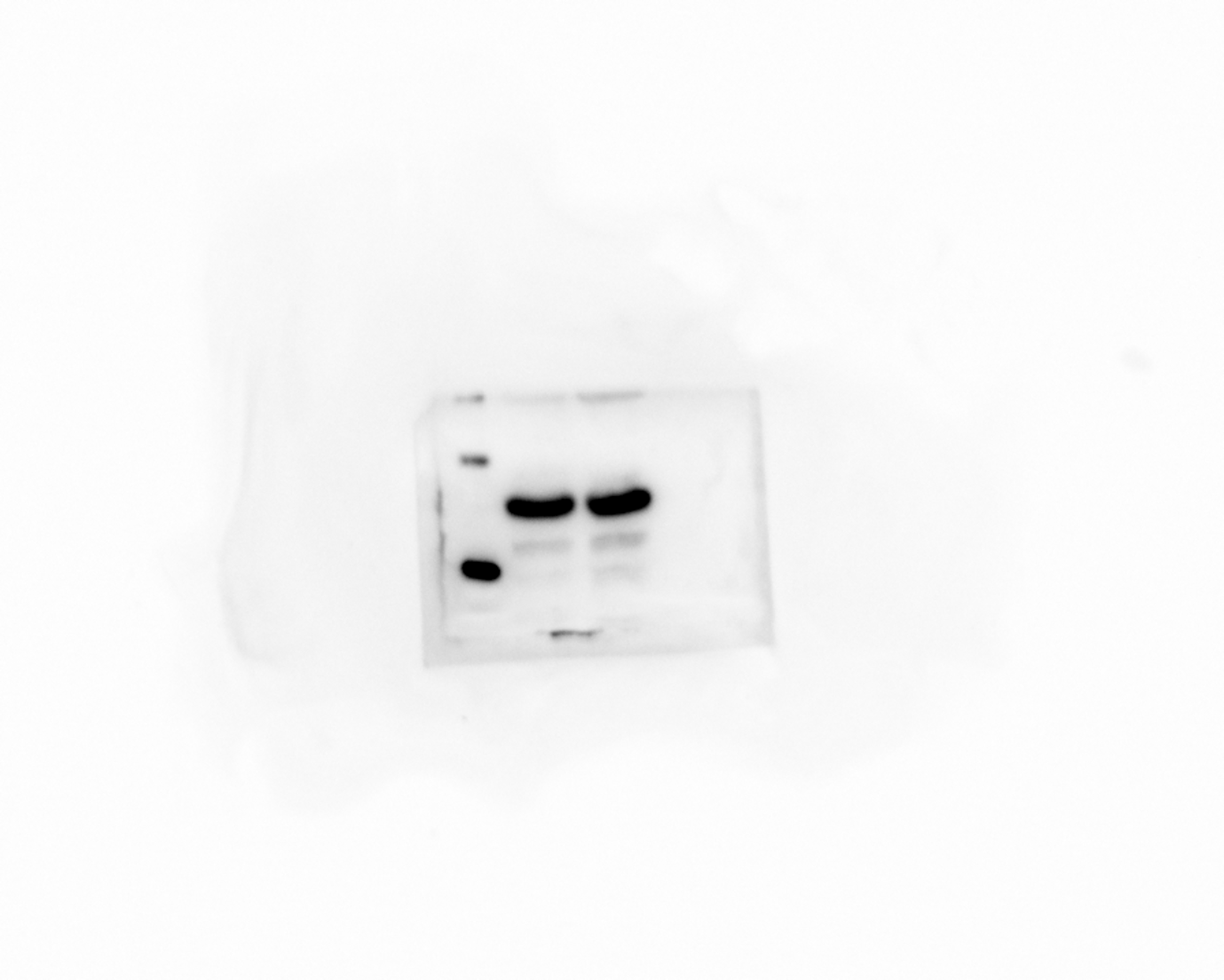

Supplement: Supplementary file 1 — Supplementary Material 1 [file 11033_2025_10940_MOESM1_ESM.zip › original versions/ΘçìσñìΣ╕Ç/σÄƒσ¢╛/╬▓-actin.png]

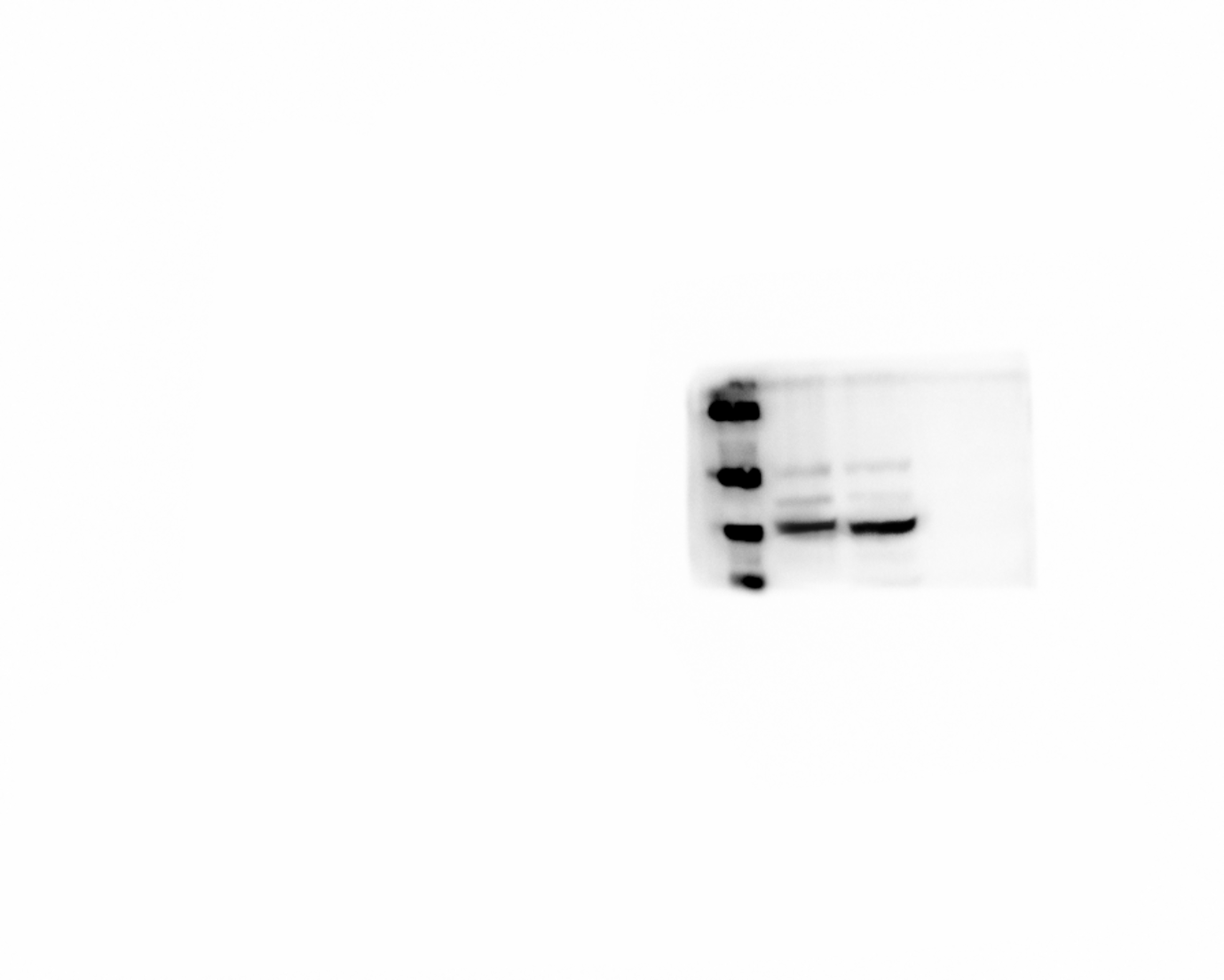

Supplement: Supplementary file 1 — Supplementary Material 1 [file 11033_2025_10940_MOESM1_ESM.zip › original versions/ΘçìσñìΣ╕Ç/σÄƒσ¢╛/METTL3.png]

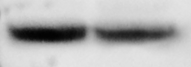

Supplement: Supplementary file 1 — Supplementary Material 1 [file 11033_2025_10940_MOESM1_ESM.zip › original versions/ΘçìσñìΣ╕Ç/μ¥íσ╕a/FTO.png]

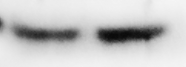

Supplement: Supplementary file 1 — Supplementary Material 1 [file 11033_2025_10940_MOESM1_ESM.zip › original versions/ΘçìσñìΣ╕Ç/μ¥íσ╕a/METTL14.png]

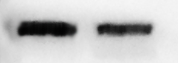

Supplement: Supplementary file 1 — Supplementary Material 1 [file 11033_2025_10940_MOESM1_ESM.zip › original versions/ΘçìσñìΣ╕Ç/μ¥íσ╕a/ALKBH5.png]

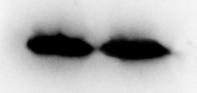

Supplement: Supplementary file 1 — Supplementary Material 1 [file 11033_2025_10940_MOESM1_ESM.zip › original versions/ΘçìσñìΣ╕Ç/μ¥íσ╕a/╬▓-actin-1.png]

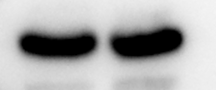

Supplement: Supplementary file 1 — Supplementary Material 1 [file 11033_2025_10940_MOESM1_ESM.zip › original versions/ΘçìσñìΣ╕Ç/μ¥íσ╕a/╬▓-actin.png]

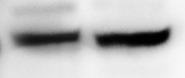

Supplement: Supplementary file 1 — Supplementary Material 1 [file 11033_2025_10940_MOESM1_ESM.zip › original versions/ΘçìσñìΣ╕Ç/μ¥íσ╕a/METTL3.png]

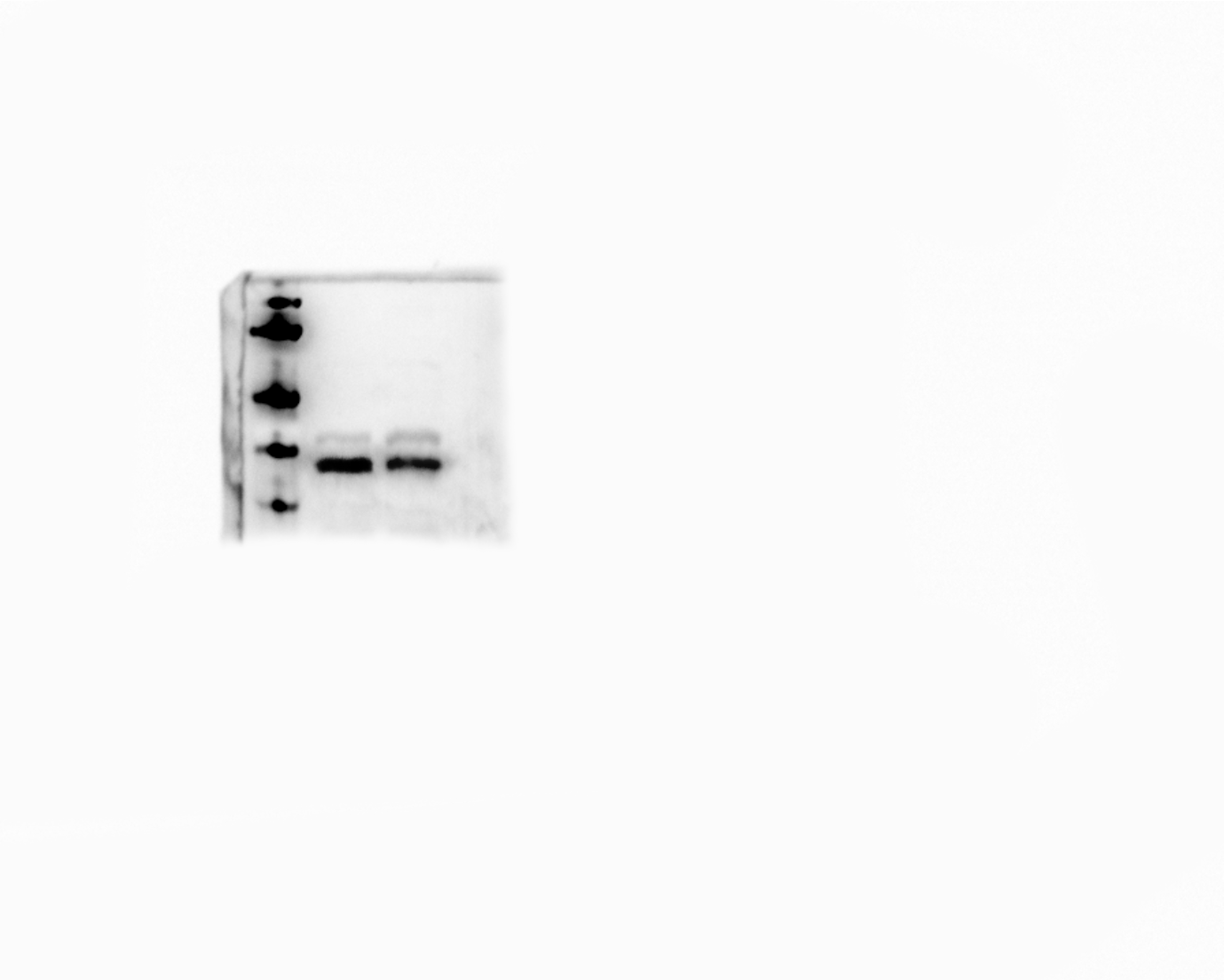

Supplement: Supplementary file 1 — Supplementary Material 1 [file 11033_2025_10940_MOESM1_ESM.zip › original versions/ΘçìσñìΣ║î/σÄƒσ¢╛/FTO.png]

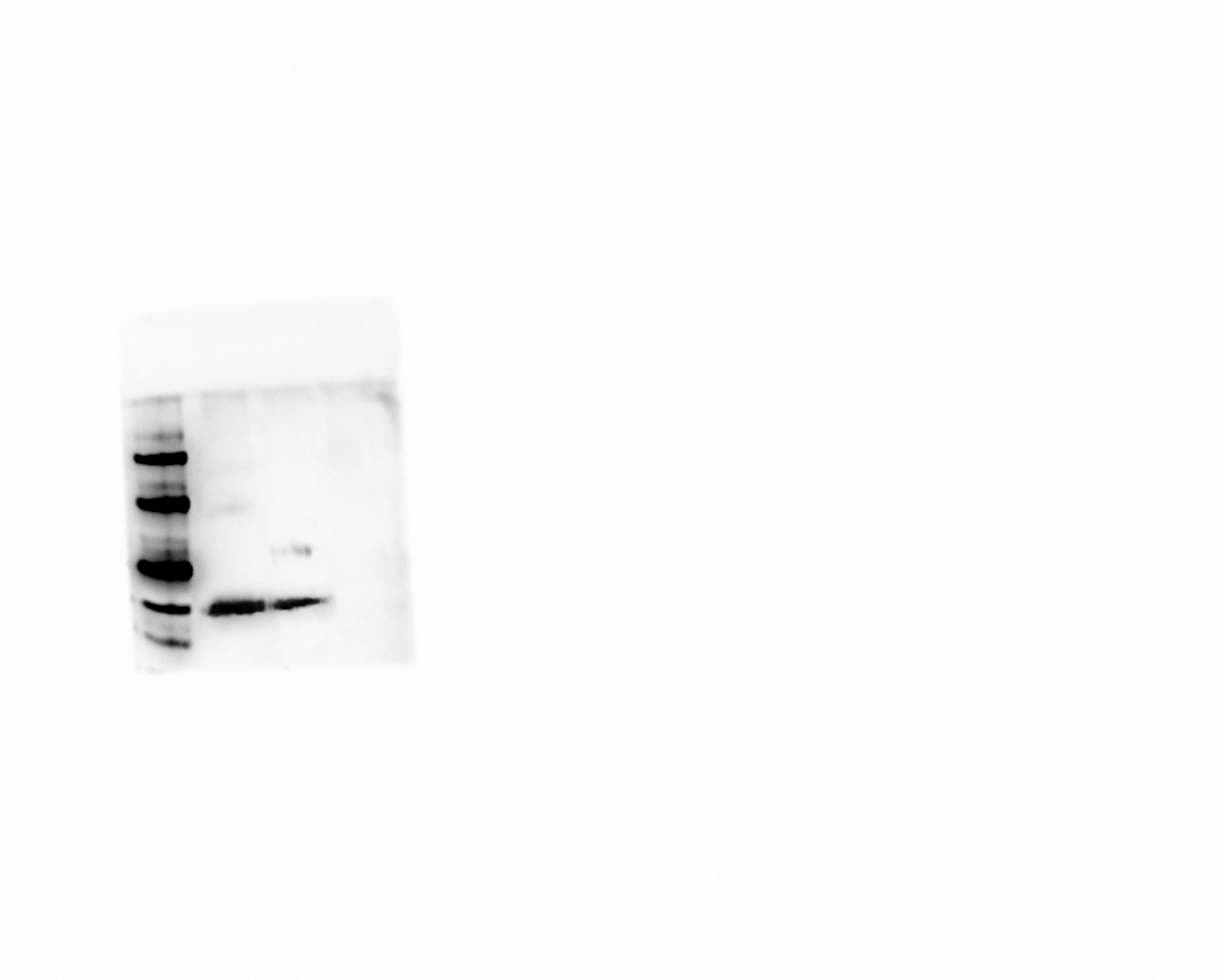

Supplement: Supplementary file 1 — Supplementary Material 1 [file 11033_2025_10940_MOESM1_ESM.zip › original versions/ΘçìσñìΣ║î/σÄƒσ¢╛/ALKBH5.png]

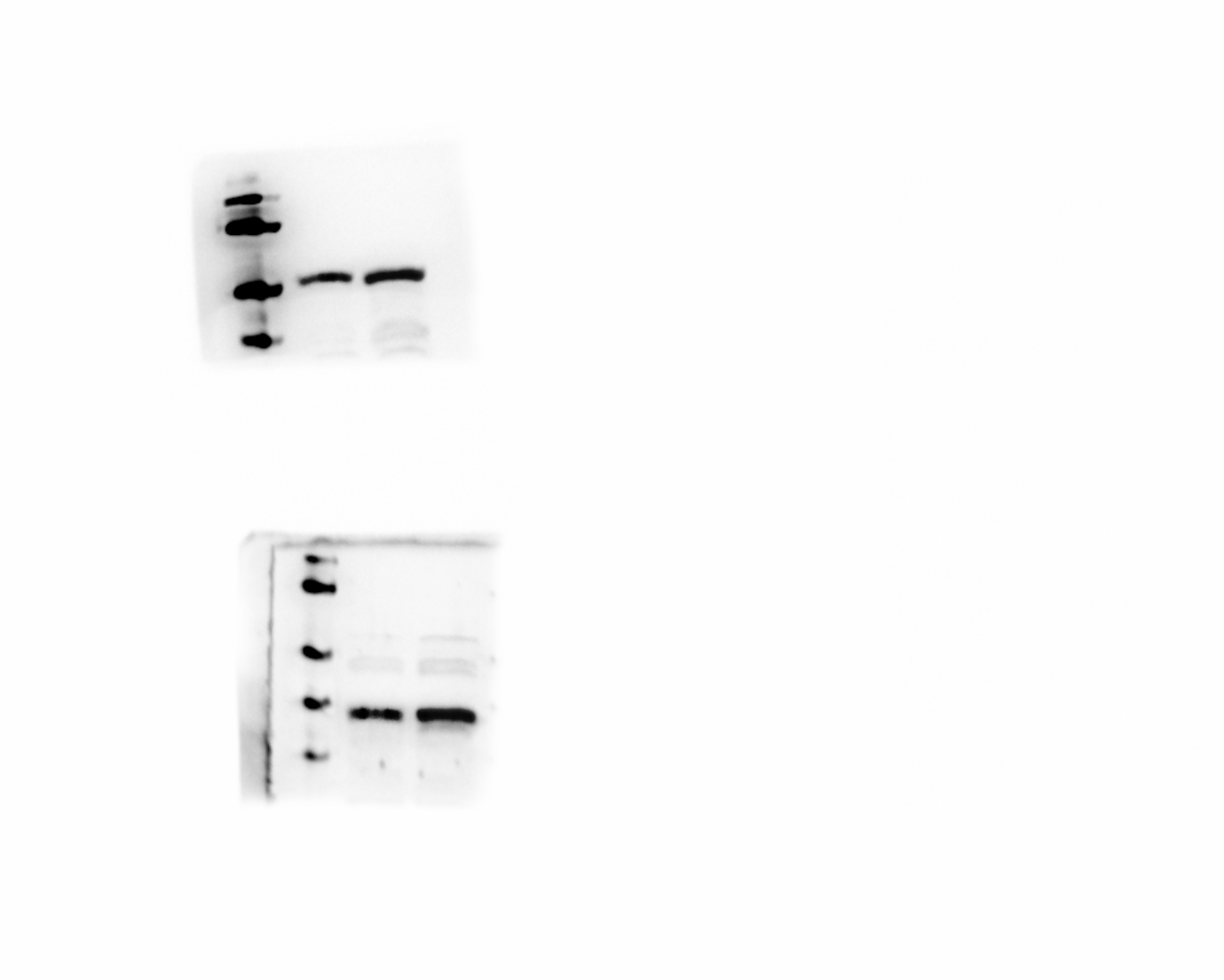

Supplement: Supplementary file 1 — Supplementary Material 1 [file 11033_2025_10940_MOESM1_ESM.zip › original versions/ΘçìσñìΣ║î/σÄƒσ¢╛/METTL3 METTL14.png]

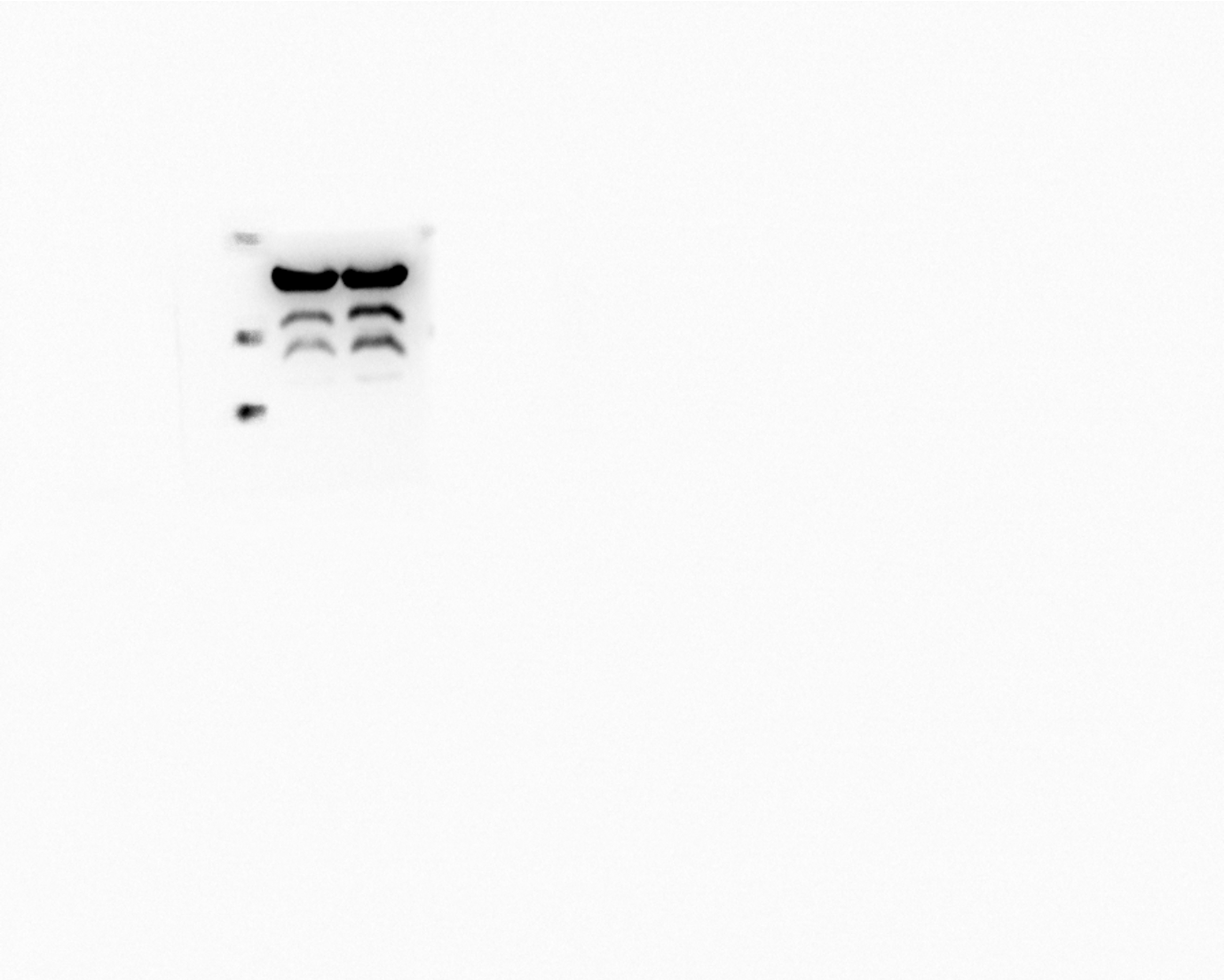

Supplement: Supplementary file 1 — Supplementary Material 1 [file 11033_2025_10940_MOESM1_ESM.zip › original versions/ΘçìσñìΣ║î/σÄƒσ¢╛/╬▓-actin-1.png]

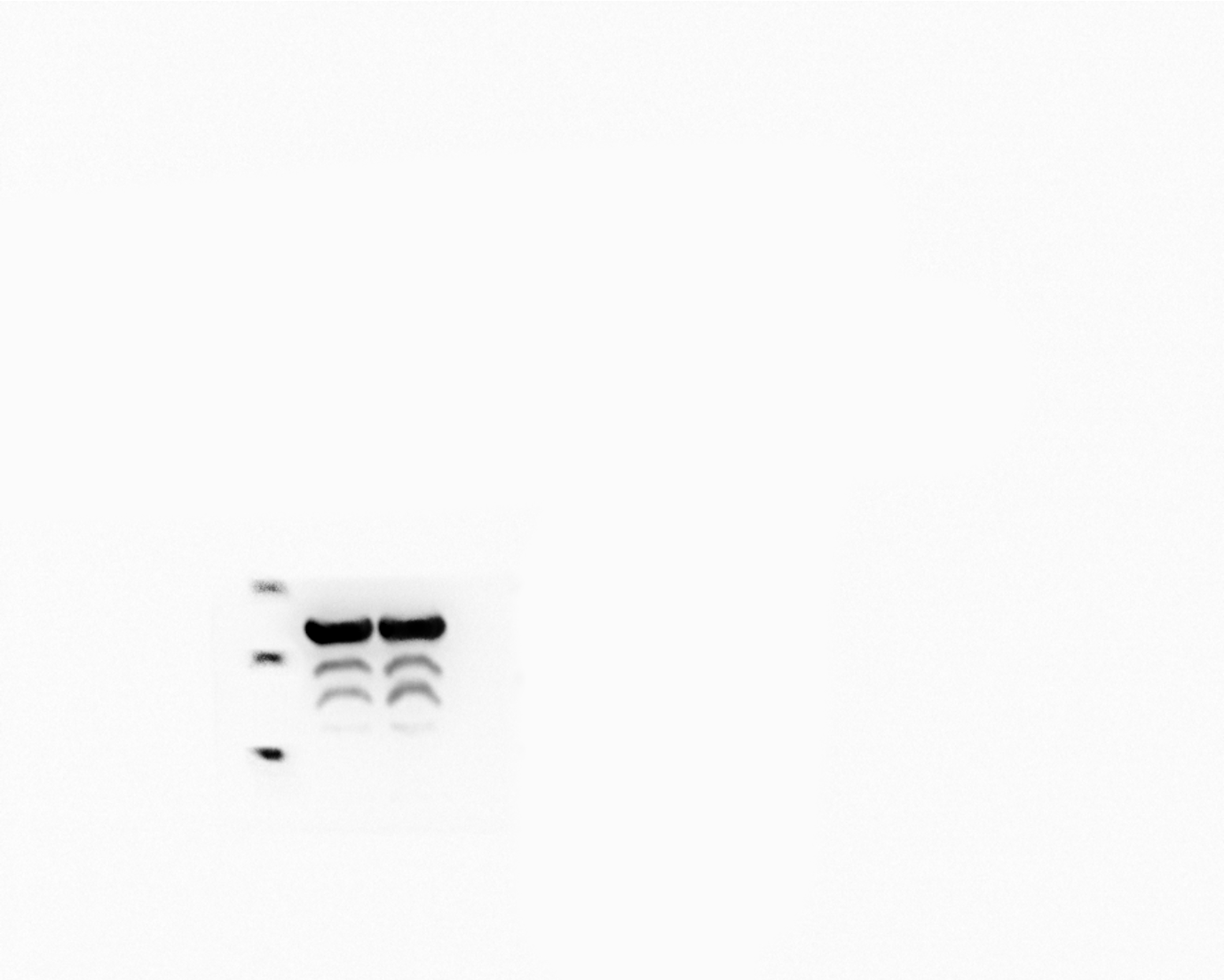

Supplement: Supplementary file 1 — Supplementary Material 1 [file 11033_2025_10940_MOESM1_ESM.zip › original versions/ΘçìσñìΣ║î/σÄƒσ¢╛/╬▓-actin.png]

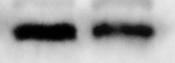

Supplement: Supplementary file 1 — Supplementary Material 1 [file 11033_2025_10940_MOESM1_ESM.zip › original versions/ΘçìσñìΣ║î/μ¥íσ╕a/FTO.png]

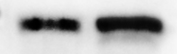

Supplement: Supplementary file 1 — Supplementary Material 1 [file 11033_2025_10940_MOESM1_ESM.zip › original versions/ΘçìσñìΣ║î/μ¥íσ╕a/METTL14.png]

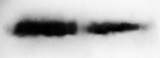

Supplement: Supplementary file 1 — Supplementary Material 1 [file 11033_2025_10940_MOESM1_ESM.zip › original versions/ΘçìσñìΣ║î/μ¥íσ╕a/ALKBH5.png]

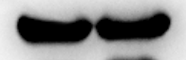

Supplement: Supplementary file 1 — Supplementary Material 1 [file 11033_2025_10940_MOESM1_ESM.zip › original versions/ΘçìσñìΣ║î/μ¥íσ╕a/╬▓-actin-1.png]

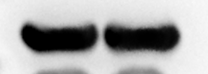

Supplement: Supplementary file 1 — Supplementary Material 1 [file 11033_2025_10940_MOESM1_ESM.zip › original versions/ΘçìσñìΣ║î/μ¥íσ╕a/╬▓-actin.png]

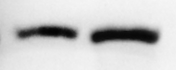

Supplement: Supplementary file 1 — Supplementary Material 1 [file 11033_2025_10940_MOESM1_ESM.zip › original versions/ΘçìσñìΣ║î/μ¥íσ╕a/METTL3.png]
